# *S*-Palmitoylation during Retinoic Acid-Induced Neuronal Differentiation of SH-SY5Y Neuroblastoma Cells

**DOI:** 10.1021/acs.jproteome.3c00151

**Published:** 2023-06-09

**Authors:** Samiksha Sardana, Anneroos E. Nederstigt, Marc P. Baggelaar

**Affiliations:** † Biomolecular Mass Spectrometry and Proteomics, Bijvoet Center for Biomolecular Research and Utrecht Institute for Pharmaceutical Sciences, University of Utrecht, Padualaan 8, Utrecht 3584 CH, The Netherlands; ‡ Netherlands Proteomics Center, Padualaan 8, Utrecht 3584 CH, The Netherlands

**Keywords:** protein *S*-palmitoylation, acyl-biotin exchange, lipid metabolic labeling, retinoic acid, SH-SY5Y differentiation, mass spectrometry-based proteomics

## Abstract

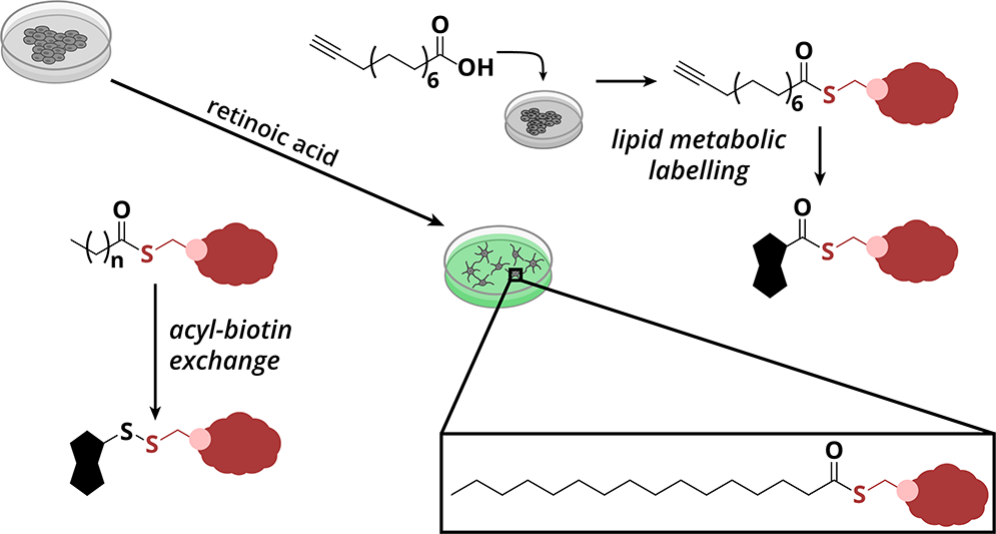

*S*-Palmitoylation is the covalent attachment of C14:0–C22:0 fatty acids (mainly C16:0 palmitate) to cysteines via thioester bonds. This lipid modification is highly abundant in neurons, where it plays a role in neuronal development and is implicated in neurodegenerative diseases, such as Alzheimer’s disease, Parkinson’s disease, and Huntington’s disease. The knowledge of *S*-palmitoylation in neurodevelopment is limited due to technological challenges in analyzing this highly hydrophobic protein modification. Here, we used two orthogonal methods, acyl-biotin exchange (ABE) and lipid metabolic labeling (LML), to identify *S*-palmitoylated proteins and sites during retinoic acid-induced neuronal differentiation of SH-SY5Y cells. We identified 2002 putative *S*-palmitoylated proteins in total, of which 650 were found with both methods. Significant changes in the abundance of *S*-palmitoylated proteins were detected, in particular for several processes and protein classes that are known to be important for neuronal differentiation, which include proto-oncogene tyrosine-protein kinase receptor (RET) signal transduction, SNARE protein-mediated exocytosis, and neural cell adhesion molecules. Overall, *S*-palmitoylation profiling by employing ABE and LML in parallel during RA-induced differentiation of SH-SY5Y cells revealed a subset of high confidence bona fide *S*-palmitoylated proteins and suggested an important role for *S*-palmitoylation in neuronal differentiation.

## Introduction

Of all human proteins, about 15% are modified by the attachment of C14:0–C22:0 fatty acids (mainly C16:0 palmitate) to cysteines via thioester bonds.^1,2^ This lipid modification is generally referred to as *S*-palmitoylation. Among other lipid post-translational modifications, *S*-palmitoylation is unique as it is the only known reversible lipid modification.^1^ It is dynamically regulated by a family of 23 DHHC protein acyltransferases (DHHC-PATs) that catalyze the attachment of fatty acids to proteins. The removal of acyl groups from proteins is catalyzed by a group of acyl-protein thioesterases, including acyl-protein thioesterases 1 and 2 (APT1/2), palmitoyl protein thioesterase 1 (PPT1), and α/β hydrolase domain containing protein 17 (ABHD17A-C).^3−5^

*S*-Palmitoylation increases membrane affinity, local hydrophobicity, and protein stability and can regulate membrane microdomain partitioning, secretion, trafficking, or protein–protein interactions.^6−8^ Moreover, *S*-palmitoylation is impaired in numerous pathological conditions, including in cancer, schizophrenia, Alzheimer’s disease (AD), and immunological diseases.^9−12^ Therefore, modulating the *S*-palmitoylation state of proteins may be a potential therapeutic strategy to treat these diseases.^1,13^

In particular, *S*-palmitoylation appears to play a key role in the central nervous system (CNS). Approximately 41% of synaptic proteins have been identified as *S*-palmitoylated, including APP, BACE1, and Huntingtin (HTT), which are involved in Alzheimer’s and Huntington’s disease (HD).^14,15^ To better understand the role of *S*-palmitoylation in the CNS in health and disease, it is crucial to study how *S*-palmitoylation is regulated in neurons.

To study *S*-palmitoylation during neuronal development, we envisioned the use of the human-derived cell line SH-SY5Y. This cell line is derived from a bone marrow biopsy of a patient with neuroblastoma and is widely used as an in vitro model for neuronal differentiation in cell-based studies because this cell line can reproduce many biochemical and morphological characteristics of neurons.^16−18^ Previous studies have determined the changes in the proteome in SH-SY5Y cells during neuronal differentiation.^18−20^ However, the role of *S*-palmitoylation in neuronal differentiation has not yet been studied in detail.

The relatively limited knowledge of *S*-palmitoylation compared to other widespread post-translational modifications such as phosphorylation and acetylation can be partly attributed to the technological challenges in analyzing *S*-palmitoylation.^21,22^ Sub-stoichiometric levels of *S*-palmitoylation, thioester lability, and the hydrophobic nature of the modification hamper the direct analysis of *S*-palmitoylated peptides by mass spectrometry.^23^ Traditionally, protein *S*-palmitoylation was analyzed in protein-specific studies using [^3^H]-palmitate metabolic labeling followed by immunoprecipitation and days to weeks of film exposure.^24^

**Figure 1:**
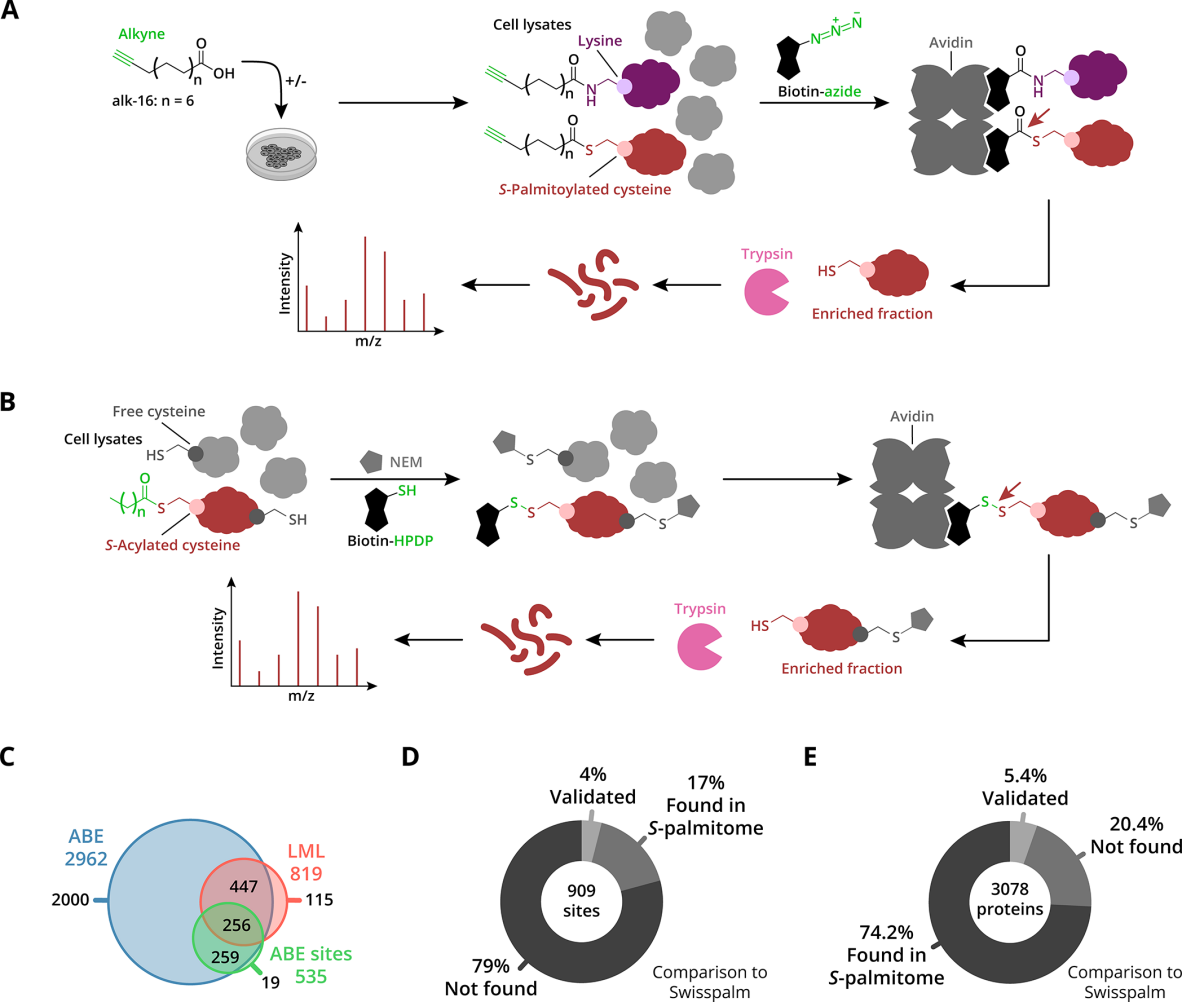
*S*-palmitoylated proteins in HEK293T cells. (A) For lipid metabolic labeling, cells are metabolically labeled with 15-hexadecynoic acid (alk-16). After cell lysis, alk-16-labeled proteins are ligated via CuAAC to biotin-azide. Next, Neutravidin affinity purification is used to enrich the biotinylated proteins, and the *S*-palmitoylated proteins are selectively released from the beads with HA. The enriched proteins are enzymatically digested, and the resulting peptides are analyzed by nano-LC–MS/MS. (B) For acyl-biotin exchange, free cysteines are blocked with NEM, followed by selective cleavage of the cysteine-acyl thioester bond with HA. Newly released thiols are reacted with a cysteine-reactive biotinylation reagent (biotin-HPDP). Neutravidin affinity purification is used to enrich the biotinylated proteins, and the *S*-acylated proteins are released from the beads with TCEP. The enriched proteins are enzymatically digested, and the resulting peptides are analyzed by nano-LC–MS/MS. (C) Venn diagram shows the overlap in protein identifications by ABE (protID and siteID) and LML. (D) Pie chart for *S*-acylation sites found by ABE compared to the SwissPalm database. (E) Pie chart shows all the proteins found by both the ABE and LML methods. Notably, 20.4% of the identified proteins are novel putative *S*-palmitoylated proteins.

Nowadays, two main indirect proteome-wide *S*-palmitoylation analysis strategies are available (Figure 1A). A “lipid-centric” approach, termed lipid metabolic labeling (LML), relies on the metabolic incorporation of an alkyne-functionalized fatty acid probe. Probe-modified proteins are ligated by copper-catalyzed alkyne–azide cycloaddition (CuAAC) to an azide–biotin conjugate, followed by enrichment by streptavidin affinity purification and analysis by mass spectrometry.^25,26^ The second strategy is a “cysteine-centric” approach termed acyl-biotin exchange (ABE) (Figure 1B). In ABE, a cysteine-reactive alkylation agent blocks all free cysteines. Next, thioesters are selectively hydrolyzed by hydroxylamine (HA) under neutral conditions. The released thiols are subsequently labeled with a cysteine-reactive biotinylation reagent, which converts previously *S*-acylated proteins to biotinylated proteins. These biotinylated proteins are then enriched by streptavidin affinity purification and analyzed by mass spectrometry.^15,27^ Both methods have their specific strengths and weaknesses (Figure S1A) but are highly complementary, which is important because indirect methods are inherently sensitive to false positive identifications. However, only few studies have performed both methods in the same system to cross-validate the results and reduce false positive identifications.^28,29^

We set out to profile *S*-palmitoylation in SH-SY5Y cells during retinoic acid-induced neuronal differentiation. Using a combination of lipid metabolic labeling and acyl-biotin exchange, we aim to (1) reduce false positive identifications by cross-validation between methods; (2) discover new *S*-palmitoylated proteins in differentiated and undifferentiated SH-SY5Y cells; and (3) gain insight into the role of *S*-palmitoylation in neuronal differentiation by monitoring steady-state *S*-palmitoylation and *S*-palmitoylation dynamics.

## Materials and Methods

### Cell Culture

HEK293T (CRL-11268, ATCC) and SH-SY5Y cells (CRL-2266, ATCC) with a passage number below 20 were cultured in full-growth medium [Dulbecco’s modified Eagle medium (DMEM, Gibco) or DMEM/F-12 (Gibco) with GlutaMAX supplement, 10% foetal bovine serum (FBS, HyClone/GE), penicillin/streptomycin (100 U/mL, 100 μg/mL, Gibco)]. Cells were maintained in a humidified atmosphere at 37 °C with 5% CO_2_.

### Neuronal Differentiation

For differentiation, SH-SY5Y cells (1.5 × 10^5^ cells/9.6 cm^2^) were plated in 10 and 15 cm plates (Greiner) 64 h before RA treatment. The full-growth medium was replaced with treatment medium [DMEM/F-12, 3% heat-inactivated FBS, penicillin/streptomycin (100 U/mL, 100 μg/mL), 10 μM RA (Sigma-Aldrich)] or with control medium [DMEM/F-12, 3% heat-inactivated FBS, penicillin/streptomycin (100 U/mL, 100 μg/mL)]; the medium was changed every 2 days. The floating cells were discarded during medium changes. Cells were harvested before treatment (*t* = 0), and after 1 (*t* = 1) and 7 days (*t* = 7) of treatment, washed twice with cold Dulbecco’s phosphate-buffered saline (DPBS, Gibco), flash-frozen and stored at −80 °C. For LML, 20 μL 25 mM 15-hexadecynoic acid (alk-16, click chemistry tools) was added to the cells (to a final concentration of 25 μM). The cells were harvested after 4 h of incubation in a humidified atmosphere at 37 °C with 5% CO_2_. The morphological changes and growth during neuronal differentiation were followed with the Incucyte ZOOM system (Sartorius/Essen Bioscience).

### Lipid Metabolic Labeling

#### Metabolic Labeling

HEK293T cells were metabolically labeled o/n with alk-16 (25 μM) in DMEM supplemented with 0.5% FBS and penicillin/streptomycin (100 U/mL, 100 μg/mL). Cells were harvested, washed twice with cold DPBS, flash-frozen and stored at −80 °C.

#### CuAAC Click Labeling and Biotin Enrichment

Frozen HEK293T cell pellets were lysed in 50 mM HEPES pH 7.5, 10 mM NaCl, 2 mM MgCl_2_, 0.5% (v/v) NP-40, 0.2% (w/v) SDS, 62.5 U/mL Benzonase nuclease (EMD Millipore corp.), 1x complete EDTA-free protease inhibitor cocktail (Roche). The protein concentration was determined by a bicinchoninic acid protein assay (BCA assay, Thermo Fisher Scientific). HEK293T cell lysates (2 mg, 1 mg/mL) were diluted with 50 mM HEPES pH 7.5, 10 mM NaCl, 2 mM MgCl_2_, and 0.5% (v/v) NP-40 to a final SDS concentration of 0.1%. Then, 120 μL of click solution [N_3_-PEG2–biotin conjugate (1 part, 10 mM stock in DMSO, Sigma-Aldrich), CuSO_4_ (2 parts, 50 mM stock in MQ, Sigma-Aldrich), TCEP (2 parts, 50 mM stock in MQ and pH 7.5, Sigma-Aldrich), and TBTA (1 part, 10 mM stock in DMSO, Fisher Scientific)] was added to the lysates and followed by incubation at RT for 1 h. To stop the reaction, 40 μL of 500 mM EDTA pH 8 was added. The samples were precipitated thrice by methanol–chloroform precipitation [4 volumes MeOH, 1 volume CHCl_3_, 4 volumes Milli-Q (MQ, Millipore)] and the pellets were washed with methanol. Protein pellets were dissolved in 150 μL of 50 mM HEPES pH 7.5, 150 mM NaCl, 2% (w/v) SDS. The samples were diluted with 50 mM HEPES pH 7.5 and 150 mM NaCl to a final SDS concentration of 0.15%. The samples were added to 25 μL NeutrAvidin beads (pre-washed with 50 mM HEPES pH 7.5, 150 mM NaCl, 0.1% SDS, Thermo Fisher Scientific) and incubated at RT for 2 h with end-to-end rotation. The beads were washed 3× with 50 mM HEPES pH 7.5, 150 mM NaCl, 0.1% SDS and 4× with 50 mM HEPES pH 7.5, 150 mM NaCl. The samples were split in two for ±hydroxylamine (HA) treatment. To the beads, 30 μL 50 mM TEA pH 7.5, 4 mM EDTA, 0.5% (w/v) Rapigest (Waters) was added, and 5 μL 7.5 M HA pH 7.5 or MQ. After incubation at RT for 1 h with end-to-end rotation, the supernatant was collected and diluted with 50 mM HEPES pH 8 to a final Rapigest concentration of 0.1%.

Frozen SH-SY5Y pellets were lysed as described above, except that 500 nM Palmostatin B (Sigma-Aldrich) was added to the lysis buffer. CuAAC reaction and precipitation with SH-SY5Y lysates (1.64 mg, 1 mg/mL) were performed as described above. SH-SY5Y protein pellets were dissolved in 62 μL 50 mM TEA pH 7.5, 150 mM NaCl, 5 mM EDTA, 4% (w/v) SDS. The samples were diluted with 50 mM TEA pH 7.5, 150 mM NaCl, 5 mM EDTA to a final SDS concentration of 2%, and then the samples were further diluted to a final SDS concentration of 0.15%. The samples were added to 21 μL NeutrAvidin beads (pre-washed with 50 mM TEA pH 7.5, 150 mM NaCl, 5 mM EDTA, 0.1% SDS) and incubated at RT for 2 h with end-to-end rotation. The beads were washed 3× with 50 mM TEA pH 7.5, 150 mM NaCl, 5 mM EDTA, 0.1% SDS and 4× with 50 mM TEA pH 7.5, 150 mM NaCl, 5 mM EDTA. The elution with HA was performed as described above. The supernatant was collected and diluted with 50 mM HEPES pH 8, 5 mM TCEP to a final Rapigest concentration of 0.1% and incubated at RT for 15 min.

#### Sample Preparation for Mass Spectrometry

The enriched samples were reacted with chloroacetamide (CAA, 20 mM) at RT for 15 min. Subsequently, the HEK293T samples were digested with LysC (0.1 μg, Wako) and trypsin (0.1 μg, Sigma-Aldrich) in 50 mM HEPES pH 8 at 37 °C o/n. The SH-SY5Y samples were digested with LysC (0.082 μg) at 37 °C for 1 h and trypsin (0.082 μg) at 37 °C o/n. The samples were quenched with TFA and centrifuged before the samples were desalted using a C18 stagetip (Empore C18 SPE disk, Supelco). Dried peptides were resuspended in 2% (v/v) formic acid and stored at −20 °C until LC–MS/MS analysis.

### Acyl-Biotin Exchange

#### Free Cysteine Alkylation

Frozen HEK293T protein pellets were lysed in 50 mM TEA pH 7.5, 10 mM NaCl, 2 mM MgCl_2_, 0.5% (v/v) NP-40, 0.2% (w/v) SDS, 62.5 U/mL Benzonase nuclease, 1x complete EDTA-free protease inhibitor cocktail and were incubated at RT for 10 min. The lysates were diluted with 1 volume 50 mM TEA pH 7.5, 10 mM EDTA, 6% (w/v) SDS and 3 volumes 50 mM TEA pH 7.5, 5 mM EDTA, 3% (w/v) SDS. The protein concentration was determined by the BCA assay. To the lysates (2 mg, 2 mg/mL), 112 μL 500 mM TCEP pH 7.5 was added, followed by incubation at RT for 30 min. Then, 35.5 μL 1 M *N*-ethyl maleimide (NEM, Sigma-Aldrich) was added to the samples, which were incubated at RT for 1 h. Again, 35.5 μL 1 M NEM was added and 568 mg urea (8 M). The samples were incubated at RT for 3 h. The samples were precipitated once by methanol–chloroform precipitation and redissolved in 400 μL 50 mM TEA pH 7.5, 150 mM NaCl, 5 mM EDTA, 2% (w/v) SDS. To the samples, 21 μL 500 mM TCEP pH 7.5 was added followed by incubation at RT for 30 min, and subsequently, 22 μL 1 M NEM was added followed by incubation at RT for 1 h. The samples were precipitated 4× by methanol–chloroform precipitation and redissolved in 400 μL 50 mM TEA pH 7.5, 150 mM NaCl, 5 mM EDTA, 2% (w/v) SDS. Frozen SH-SY5Y pellets were lysed as described above, except that 500 nM Palmostatin B was added to the lysis buffer. The SH-SY5Y lysates (1.1 mg, 1 mg/mL) were reduced and alkylated as described above.

#### Biotin Labeling and Enrichment

HEK293T samples were split in two for ±HA treatment. To the samples, 200 μL 5 mM HPDP-biotin (in DMF/MQ = 2:1, Cayman Chemical) was added and 62 μL 7.5 M HA pH 7.5 or MQ. The samples were incubated at RT for 90 min. The samples were precipitated thrice by methanol–chloroform precipitation and redissolved in 75 μL 50 mM TEA pH 7.5, 150 mM NaCl, 5 mM EDTA, 2% (w/v) SDS. The samples were diluted with 50 mM TEA pH 7.5, 150 mM NaCl, and 5 mM EDTA to a final SDS concentration of 0.1%. The samples were added to 25 μL NeutrAvidin beads (pre-washed with 50 mM TEA pH 7.5, 150 mM NaCl, 5 mM EDTA, 0.1% SDS) and incubated at RT for 2 h with end-to-end rotation. The beads were washed 3× with 50 mM TEA pH 7.5, 150 mM NaCl, 5 mM EDTA, 0.1% SDS and 4× with 50 mM TEA pH 7.5, 150 mM NaCl, 5 mM EDTA. To the beads, 70 μL 50 mM TEA pH 7.5, 50 mM TCEP, 5 mM EDTA, 0.1% (w/v) Rapigest was added. After incubation at RT for 30 min with end-to-end rotation, the supernatant was collected and diluted with 230 μL 50 mM HEPES pH 8.

The SH-SY5Y samples were biotinylated as described above. SH-SY5Y protein pellets after precipitation were dissolved in 20.5 μL 50 mM TEA pH 7.5, 150 mM NaCl, 5 mM EDTA, 4% (w/v) SDS. The samples were diluted with 50 mM TEA pH 7.5, 150 mM NaCl, and 5 mM EDTA to a final SDS concentration of 2%, and then the samples were further diluted to a final SDS concentration of 0.1%. The samples were added to 14 μL NeutrAvidin beads, and the enrichment was performed as described above.

#### Sample Preparation for Mass Spectrometry

The enriched samples were reacted with CAA (25 mM) at RT for 20 min. Subsequently, the HEK293T samples were digested with LysC (0.1 μg) at 37 °C for 30 min and trypsin (0.1 μg) at 37 °C overnight. The SH-SY5Y samples were digested with LysC (0.054 μg) at 37 °C for 1 h and trypsin (0.054 μg) at 37 °C overnight. The samples were quenched with TFA and centrifuged before the samples were desalted using a C18 stage tip. Dried peptides were resuspended in 2% (v/v) formic acid and stored at −20 °C until LC–MS/MS analysis.

### Proteomics

#### Sample Preparation for Total Proteome

SH-SY5Y lysates (20 μg, 1 mg/mL, from the ABE experiment) were reduced with 5 μL 100 mM DTT (in 50 mM ammonium bicarbonate pH 8, Sigma-Aldrich) at RT for 45 min and alkylated with 3.9 μL 300 mM iodoacetamide (in 50 mM ammonium bicarbonate pH 8, Sigma-Aldrich) at RT for 30 min in the dark. The samples were prepared and digested according to the manufacturer’s instructions. As follows, 2× SDS solubilization buffer (100 mM TEAB pH 7.5, 10% (w/v) SDS) was added to the samples, followed by 12% phosphoric acid (1:10) and 6× S-trap protein binding buffer (100 mM TEAB pH 7.1, 90% MeOH). The samples were loaded on the S-trap column (PROTIFI) and washed 5× with S-trap binding buffer. Digestion buffer (1:25 trypsin in 50 mM ammonium bicarbonate pH 8) was added to the column. After incubation at 47 °C for 1 h without shaking, peptides were eluted sequentially with 40 μL 50 mM ammonium bicarbonate pH 8, 40 μL 0.2% formic acid and 35 μL 50% acetonitrile, 0.2% formic acid. Dried peptides were resuspended in 2% (v/v) formic acid and stored at −20 °C until LC–MS/MS analysis.

#### Mass Spectrometry Analysis

Samples were analyzed on a nanospray UHPLC system Ultimate 3000 (Thermo Fisher Scientific) coupled to an Orbitrap Exploris 480 mass spectrometer (Thermo Fisher Scientific) in data-dependent acquisition mode. Peptides were loaded onto Acclaim Pepmap 100 C_18_ trap column (5 × 0.3 mm, 5 μmm Thermo Fisher Scientific) in solvent A (0.1% formic acid) and separated on an analytical column (Poroshell 120 EC C_18_, 50 cm × 75 μm, 2.7 μm, Agilent Technologies) with a flowrate of 300 nL/min. For the HEK293T peptides from the LML experiment, a 115 min gradient was used: 9% solvent B (0.1% formic acid in 80% acetonitrile) for 1 min, 9–13% for 1 min, 13–44% in 95 min, 44–99% in 3 min, 99% for 4 min, 99–9% for 1 min, and 9% for 10 min. For the SH-SY5Y peptides from the LML experiment, a 85 min gradient was used: 9% solvent B for 1 min, 9–13% for 1 min, 13–44% in 65 min, 44–99% in 3 min, 99% for 4 min, 99–9% for 1 min, and 9% for 10 min. For the peptides from the ABE experiment, the gradient of 115 min was used. For the peptides from the total proteome experiment, a gradient of 175 min was used: 9% solvent B for 1 min, 9–13% for 1 min, 13–44% in 155 min, 44–99% in 3 min, 99% for 4 min, 99–9% for 1 min, and 9% for 10 min. MS1 scans were performed at a resolution of 60,000 between 375 and 1600 *m*/*z* after reaching the normalized AGC target with automatic injection time every second. Top intense precursors were fragmented with normalized collision energy of 28% and 16s dynamic exclusion time. HCD fragmentation was performed on precursors at a resolution of 30,000.

#### MaxQuant Search

Data were processed with MaxQuant version 2.0.1.0 or 2.0.3.0, and the MS/MS spectra were searched against the human Uniprot database (version November 2022, UP000005640) using the Andromeda search engine. All files were analyzed by using the built-in label-free quantification (LFQ) algorithm separated based on parameter groups. Cysteine carbamidomethylation, methionine oxidation, and protein N-terminal acetylation were set as variable modifications. For ABE, cysteine modified by NEM was additionally used as a variable modification. Enzyme specificity was set to LysC and trypsin. Other parameters were used as pre-set in the software.

#### Data Processing

Data from the “proteinGroups.txt” file was processed with Excel 2016 and Perseus version 1.6.14.0. Proteins with zero cysteines were filtered from the ABE and LML data. Ubiquitin-activating and ubiquitin conjugating enzymes and ubiquitin ligases are enzymes with thioesters as catalytic intermediates and were thus also removed from the ABE data. Protein hits with unique peptides ≤1 were filtered from all data. In Perseus, potential contaminants, reverse and only identified by sites were filtered. LFQ intensities were log2 -transformed. After grouping the replicates, the data was filtered to keep 3 valid values out of 3 (2 valid values out of 2 for HEK293T ABE) per protein. For ABE and LML, the data was checked for normal distribution before imputing missing values (width 0.3, down shift 1.8). A Student’s *t*-test (permutation-based, 250 permutations, FDR = 0.01, and *s*_0_ value = 0.5) was performed to compare with and without HA samples. Proteins significantly enriched in HA-treated samples were defined as putative *S*-palmitoylated proteins. The protein hit lists were compared to the SwissPalm database (www.swisspalm.org).^2^ For the neuronal differentiation experiment, this list of *S*-palmitoylated proteins was used for further data processing. The median was subtracted to compensate for systematic measurement effects. Missing values were imputed as described above, and the Student’s *t*-test was performed to compare with and without RA samples. For total proteome, after all the filtering, the median was subtracted, missing data was imputed, and the Student’s *t*-test was performed as described above. Venn diagrams were created on meta-chart (www.meta-chart.com). Gene ontology (GO) analysis was performed with panther (www.pantherdb.org)^30^ and visualized in RStudio version 2022.07.2 + 576 using R version 4.1.0.

Data from the “Carbamidomethyl C Sites.txt” file was processed with Excel 2016. Ubiquitin-activating and ubiquitin-conjugating enzymes and ubiquitin ligases were removed from the data. Potential contaminants and reverse were removed. Additionally, the data was filtered to keep 3 valid values out of 3 per protein for the +HA samples for SH-SY5Y ABE and 2 valid values out of 2 for HEK293T ABE. RAW intensities were log2-transformed, and the fold change was calculated. The hits were defined as confident site-containing peptides if, in all three −HA replicates, the intensity was 0 or fold change ≥2. Additionally, a hit was kept if the localization probability ≥0.75 in 2 out of 3+ HA replicates. The position of the site in the site-containing peptides was compared to the SwissPalm database. MS/MS spectra were visualized using in-house software. Data were further analyzed and visualized with Graphpad Prism 9 and Adobe Illustrator 2023.

### Acyl-PEG Exchange Assay

#### Hydroxylamine Cleavage and mPEG-Maleimide Alkylation

Frozen SH-SY5Y pellets were lysed, reduced with TCEP (761 μg, 2 mg/mL), and alkylated with NEM as described above. Samples were divided into two for ± HA treatment. Samples were treated with either 6 μL of 7.5 M HA to a final concentration of 1 M or with 6 μL of MQ and incubated at RT for 90 min, after which they were precipitated once by methanol–chloroform precipitation. Samples were dissolved in 50 mM TEA pH 7.5, 150 mM NaCl, 5 mM EDTA, 4% (w/v) SDS. The samples were diluted with 50 mM TEA pH 7.5, 150 mM NaCl, and 5 mM EDTA to a final SDS concentration of 2%, and then the samples were further diluted to a final SDS concentration of 0.1%. Samples were divided in two once again. 3.9 μL of 500 mM TCEP (final concentration 10 mM) was added, and samples were incubated at RT for 15 min. 11.82 μL of 43.5 mM of either mPEG-10k (Sigma-Aldrich) or NEM was added to a final concentration of 2.5 mM, and incubated at RT for 1 h before a final precipitation. Samples were dissolved in 50 mM TEA pH 7.5, 150 mM NaCl, 5 mM EDTA, 4% (w/v) SDS.

#### Western Blotting

To 12.5 μL (50 μg) of each sample, 4.2 μL of 4× XT sample buffer (Bio-Rad) supplemented with 10% 2-mercaptoethanol was added, followed by 5 min of heating at 95 °C. 15 μL (45 μg) of each sample was loaded on 4–12% Criterion XT Bis-Tris Protein Gels (Bio-Rad) and separated by SDS-PAGE, after which they were transferred onto 0.2 μM PVDF membranes (Bio-Rad) to be analyzed by western blotting. Primary antibodies used were anti-NCAM2 (1:2,000, Santa Cruz Biotechnology) and anti-Actin (1:5,000, Sigma-Aldrich). Secondary antibodies used were anti-Mouse IgG, HRP-linked (1:10,000, Cell Signaling) and anti-Rabbit IgG, HRP-linked (1:10,000, Cell Signaling). Proteins were detected with Pierce ECL Plus Western Blotting Substrate (Thermo Fisher Scientific) and an AI600 Imager (GE Healthcare). Images were further processed and visualized with ImageJ version 1.52 and Adobe Illustrator 2023. For proteome analysis, 30 μL (15 μg) of SH-SY5Y whole cell lysate of each sample was analyzed by SDS/PAGE followed by western blotting as described above with minor changes. Primary antibodies used were anti-NCAM2 (1:2000) and anti-Actin (1:5000). Secondary antibodies used were polyclonal Goat Anti-mouse Immunoglobulin/HRP (1:2000, Agilent) and anti-Rabbit IgG, HRP-linked (1:10,000).

## Results and Discussion

### *S*-Palmitoylation Profiling in HEK293T Cells by Orthogonal Methods

Method sensitivity and the reduction of false positives for ABE strongly relies on the efficiency of cysteine capping before thioester hydrolysis. Recently, 4,4′-dithiodipyridine (DTDP) capping was introduced as an additional capping step to efficiently block all free cysteines in cell lysates.^27^ However, DTDP capping is not compatible with the method that we envisioned, which is a dual *S*-acylation site and complete protein detection by selective labeling of previously *S*-acylated cysteines with chloroacetamide (CAA) after TCEP-mediated release from neutravidin beads (Figure 1B and Figure S2A). DTDP blocking is reversible under reducing conditions, which are required for protein release from neutravidin beads, and is therefore not compatible with this strategy. Our alternative approach involves capping cysteines twice with *N*-ethylmaleimide (NEM) after TCEP-mediated reduction of cysteines with various oxidation states. This method allows for nearly complete blockage of “free” cysteines while the capping remains stable under reducing conditions.

Using the highly efficient double capping procedure, we explored a LFQ proteomics methodology for ABE-based dual *S*-acylation site and complete protein analysis in HEK293T cells. LFQ was applied as its robustness for *S*-acylation profiling was previously shown by Zhou et al. by comparing it to a SILAC quantification.^27^ As a result, 2962 hydroxylamine (HA)-sensitive proteins were detected, which is comparable to the currently most sensitive low-background ABE method reported by Zhou et al.,^27^ and 909 CAA-labeled sites were discovered (Figures 1C,D and S2B). Amongst the identified proteins are well-known and validated *S*-palmitoylated proteins, such as GTPases HRAS and NRAS, protein scribble homolog (SCRIB), and Flotillin-1 (FLOT1). In addition, 16 of the 23 known *S*-palmitoyl transferases (ZDHHCs) were identified, indicating the expression of most ZDHHC family members in HEK293T cells. The ZDHHCs can be detected through either their catalytic cysteine or *S*-palmitoylated cysteines residing outside the catalytic domain. *S*-acylation site identification revealed *S*-acylation of 17 non-catalytic cysteines in eight ZDHHCs (Figure S2C,D). Of these, 12 cysteines are novel sites, and five sites in ZDHHC5 and ZDHHC6 have been validated by site-directed mutagenesis and gel-based *S*-palmitoylation analysis.^31,32^

Despite TCEP reduction before NEM capping of cysteines, incomplete capping of cysteines involved in disulfide bridges may result in false positive identifications of *S*-acylation sites. The Uniprot database was used to gain insight into the number of CAA-labeled sites that are involved in disulfide bridge formation. We found 8.25% (75 out of 909) of the CAA-labeled cysteines to be involved in disulfide bridge formation, which includes Cys73 of thioredoxin. This particular cysteine residue is known to be modified by both *S*-glutathionylation and *S*-palmitoylation.^33^ Although cysteines involved in disulfide bond formation may have a higher probability of being a false positive, they can still be genuine *S*-palmitoylation sites.

ABE is an indirect method for the detection of *S*-acylated proteins; therefore, we aimed to compare the proteins detected by this method with an orthogonal strategy using palmitic acid alkyne (alk-16). We adopted the alk-16 labeling strategy coupled with mass spectrometry proteomics from Thinon et al.^28^ A key feature of this strategy reported by Thinon et al. is the on-bead thioester hydrolysis step followed by in-solution digestion instead of on-bead digestion (Figure 1A). This step not only ensures the selection of proteins that are acylated through a thioester bond but also reduces neutravidin contaminants during mass spectrometry analysis, thereby increasing the sensitivity of this methodology.

By labeling with alk-16 in HEK293T cells, we identified 819 significantly enriched proteins in the HA-treated samples compared to non-HA-treated samples (Figures 1C and S3A). In addition, using vehicle (DMSO) as a control, we show the requirement of alk-16 for enrichment of *S*-palmitoylated proteins (Figure S3B,C). A total of 703 proteins were found with both ABE and LML (Figure 1C), providing high confidence that these proteins are bona fide *S*-palmitoylated proteins. *S*-palmitoylated proteins that are only identified by ABE can originate from slow (*S*-palmitoylation) turnover and/or *S*-acylation by lipids other than palmitic acid. Comparison of the relative mass spectrometry-based LFQ intensities of the 703 proteins identified by both methods shows a clear correlation but higher signal intensities for proteins detected by ABE (Figure S3D). This is in-line with the assumption of sub-stoichiometric incorporation levels of alk-16 compared to endogenous *S*-acylation.^34^ Comparison of the 3078 identified proteins with the SwissPalm database reveals that 74% have been found before in palmitoyl-proteome studies and 5% have been validated (Figure 1E). Of note, it is important to exercise caution when using SwissPalm as a reference database because it comprises datasets from multiple studies, each with their own statistical analysis. This likely results in a high FDR.

The ABE experiment was performed in duplicate, which limits the statistical power of this experiment. However, the analysis reveals that a combination of LML and ABE still allows for deep palmitoyl-proteome profiling, and identifies a subset of high confidence bona fide *S*-palmitoylated proteins that can be used for future reference. We next set out to use these methods to study *S*-palmitoylation in neuronal differentiation.

### Retinoic Acid-Induced Differentiation of SH-SY5Y Cells

**Figure 2:**
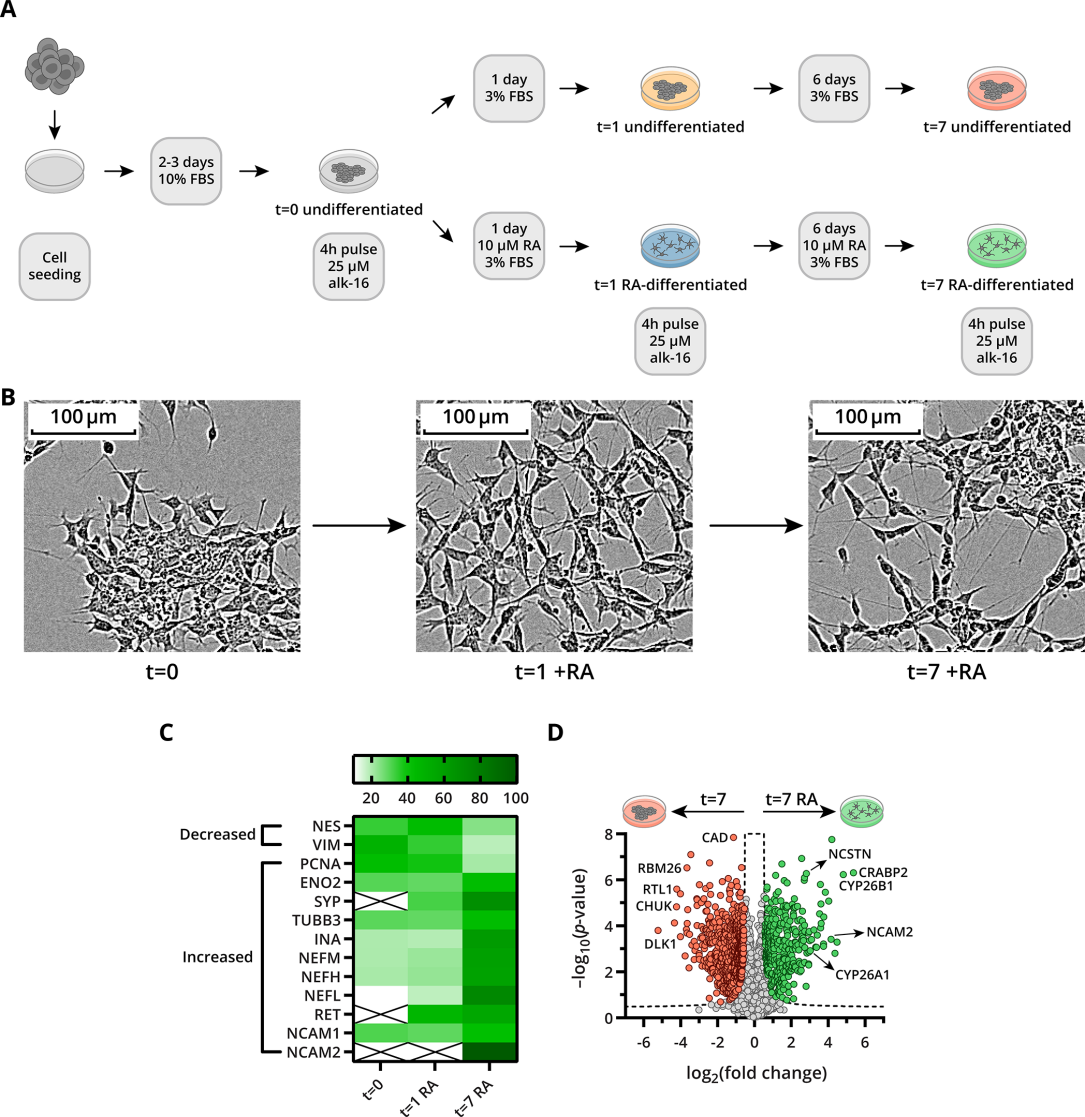
Morphology and proteome changes in SH-SY5Y cells during RA-induced neuronal differentiation. (A) Experimental workflow for neuronal differentiation of SH-SY5Y cells with RA over a 7 day period. (B) Phase contrast microscopy images of morphological changes in SH-SY5Y cells during RA-induced neuronal differentiation over 7 days (magnification: 20×). (C) Heatmap of the relative abundance of marker proteins. Each protein abundance value sums to 100 across the three conditions. (D) Volcano plot of the LFQ proteomics analysis of proteins in SH-SY5Y cells at *t* = 7 (±RA). Proteins downregulated during differentiation are pink, and upregulated proteins are green. Dashed line represents the Student’s unpaired *t*-test significance cut-off (FDR 0.01, S0 0.5, and *n* = 3 biological replicates).

The neuroblastoma cell line SH-SY5Y was differentiated to a neuronal-like phenotype by well-established methods using retinoic acid (RA).^16,18−20^ SH-SY5Y cells were stimulated with or without 10 μM RA in low serum conditions for 7 days (Figure 2A). Samples were analyzed before RA stimulation (*t* = 0), after 1 day (*t* = 1), and after 7 days (*t* = 7) of RA treatment. Multiple time points were taken because time-dependent changes during neuronal differentiation have been identified previously.^18^ Clear morphological changes were observed between RA-treated and vehicle-treated cells (Figures 2B and S4A). Undifferentiated SH-SY5Y cells had few, short processes and grew in clusters, while differentiated cells became morphologically similar to primary neurons with long, pronounced processes.

The total proteome of RA-stimulated and vehicle-treated cells at *t* = 0, *t* = 1, and *t* = 7 was analyzed by mass spectrometry. In total, 3462 proteins were quantified in RA-treated and vehicle-treated SH-SY5Y cells. Known markers of neuronal differentiation, including neurofilaments (NEFs), tubulin beta 3 class III (TUBB3), enolase 2 (ENO2), neural cell adhesion molecule 2 (NCAM2), and synaptophysin (SYP), showed a marked increase in protein abundance after 7 days of differentiation (Figure 2C).^19,35−38^ Levels of Nestin (NES) and vimentin (VIM) are known to decrease upon neuronal differentiation and were indeed reduced in protein abundance after 7 days of RA stimulation.^35^ These changes in the proteome became more pronounced over time. Analysis of the relative abundance of proteins between undifferentiated and differentiated cells at *t* = 1 revealed significant upregulation of 194 proteins and downregulation of 130 proteins (Figure S4B), while after 7 days of RA stimulation, 349 proteins are upregulated and 553 proteins are downregulated (Figure 2D). The analysis of the total proteome revealed the presence of four known *S*-acyl protein thioesterases, namely ABHD10, LYPLA1, LYPLA2, and PPT1. Although the differences in protein abundance between the time points for these proteins are small, the abundance of PPT1, ABHD10, and LYPLA2 appears to increase after 7 days of RA stimulation (Figure S5A–D).

GO term enrichment analysis using PantherDB revealed that RA metabolic processes are already upregulated after 1 day.^39^ A strong increase was observed in levels of cytochromes P450 26A1 and 26B1 (CYP26A1 and CYP26B1), cellular retinoic acid-binding protein (CRABP2), and retinol-binding protein (RBP1) (Figures 2D and S4B,C).^20^ Other rapidly upregulated processes (within 1 day) were lipid metabolic process, nucleotide metabolic process, and organelle organization. After 7 days of differentiation, proteome remodeling toward a neuronal phenotype was clearly visible by the overrepresentation of biological processes including neurofilament bundle assembly, postsynaptic intermediate filament cytoskeleton organization, and pyramidal neuron differentiation in RA-treated cells (Figure S4C). Moreover, several proteins important for neuronal differentiation, including copper-transporting ATPase 1 (ATP7A), apoptosis regulator Bcl-2 (BCL2), integrin alpha-1 (ITGA1), integrin beta-1 (ITGB1), microtubule-associated proteins 1A and 1B (MAP1A and MAP1B), NCAM2, nicastrin (NCSTN), and proto-oncogene tyrosine-protein kinase receptor (RET), were elevated after 7 days of RA stimulation (Figure 2D). In line with reduced proliferation of neurons, proteins involved in DNA and protein synthesis and cell cycle regulation were downregulated upon neuronal differentiation (Figures 2D and S4D). In conclusion, RA treatment of SH-SY5Y induced differentiation toward a neuronal phenotype by increasing the expression of proteins involved in neuronal differentiation, while proteins involved in the replication and proliferation machinery were downregulated.

### *S*-Palmitoylation Profiling during Neuronal Differentiation of SH-SY5Y Cells

**Figure 3:**
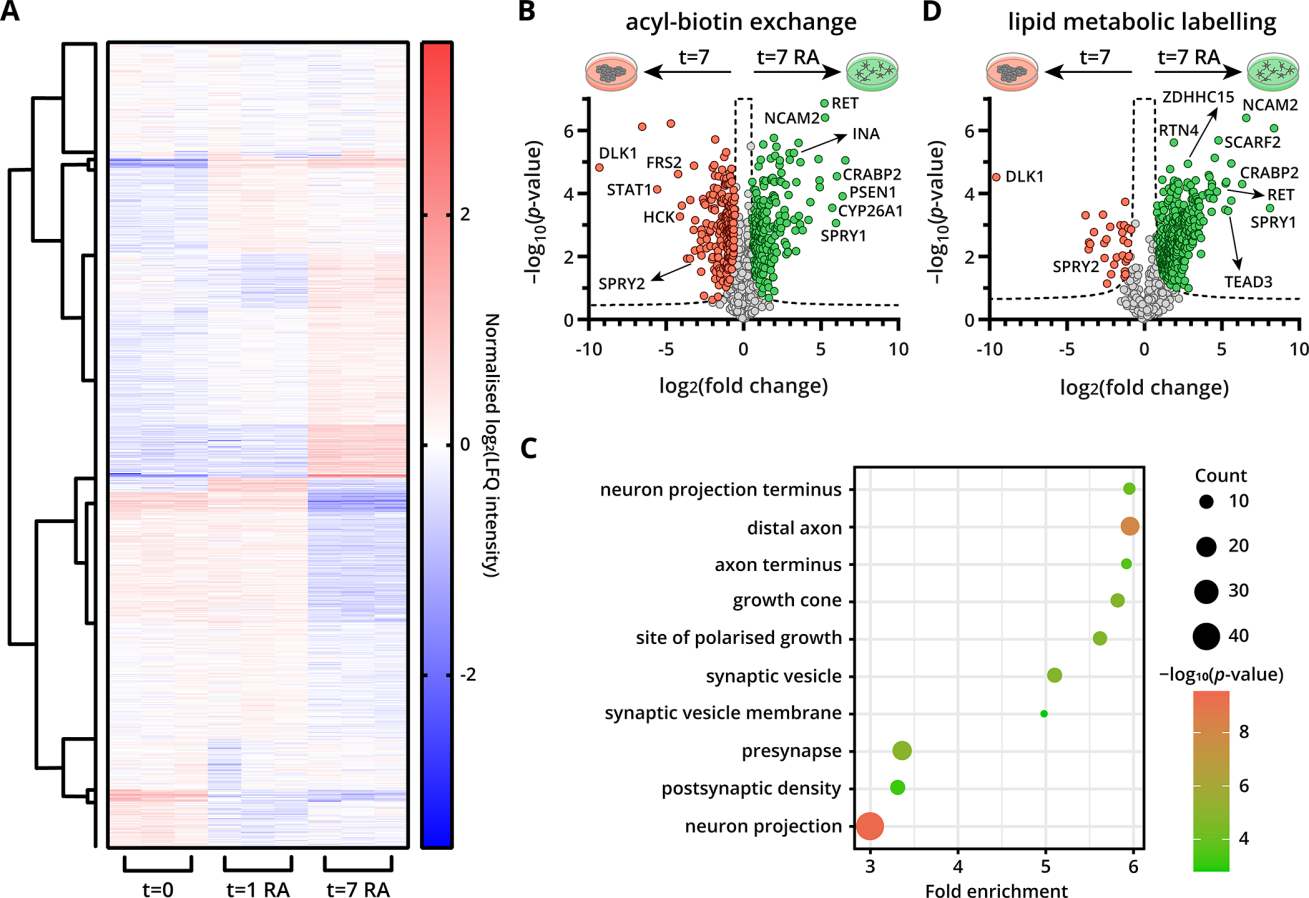
*S*-palmitoylation profiling of SH-SY5Y cells during RA-induced neuronal differentiation. (A) Heatmap of the relative abundance of 1151 *S*-acylated proteins found in all three time points. (B) Volcano plot of the LFQ proteomics analysis of *S*-acylated proteins in SH-SY5Y cells at *t* = 7 enriched with the ABE workflow (±RA). Proteins decreased in *S*-palmitoylated protein abundance during differentiation are pink, and proteins increased are green. Dashed line represents the Student’s unpaired *t*-test significance cut-off (FDR 0.01, S0 0.5, and *n* = 3 biological replicates). (C) GO cellular component analysis for proteins increased in *S*-palmitoylated protein abundance at *t* = 7 (FDR <0.05, ABE). (D) Volcano plot of the LFQ proteomics analysis of alk-16-labeled proteins in SH-SY5Y cells at *t* = 7 enriched with LML workflow (±RA). Proteins decreased in *S*-palmitoylated protein abundance during differentiation are pink, and proteins increased are green. Dashed line represents the Student’s unpaired *t*-test significance cut-off (FDR 0.01, S0 0.5, and *n* = 3 biological replicates).

*S*-palmitoylation in SH-SY5Y cells was monitored by ABE before RA-induced differentiation (*t* = 0) and after 1 day (*t* = 1) and 7 days (*t* = 7) of stimulation (Figure S6A–E). In total, 1931 *S*-acylated proteins and 475 *S*-acylation sites were identified. The abundance profiles of 1151 proteins that were identified in all three time points are displayed in a heatmap (Figure 3A). A tight clustering among biological replicates was observed with a clear difference between undifferentiated and differentiated cells, indicating that RA impacts *S*-palmitoylated protein abundance. This clear difference between the time points and the reproducibility of the replicates was also reflected by a principal component analysis (Figure S6F).

Changes in the abundance of *S*-palmitoylated proteins were already observed after 1 day of RA-induced neuronal differentiation (Figure S6G). Interestingly, the differences were more pronounced after 7 days of differentiation. After 7 days, the abundance of *S*-palmitoylated proteins increased for 211 proteins and decreased for 158 proteins (Figure 3B). After 1 day of differentiation, *S*-palmitoylated protein abundance was increased and decreased for 165 and 93 proteins, respectively (Figure S6G). For multiple proteins that play a key role in neuronal differentiation, the *S*-palmitoylated protein abundance was significantly elevated in differentiated cells, including internexin neuronal intermediate filament protein alpha (INA), fibroblast growth factor receptor substrate 2 (FRS2), hematopoietic cell kinase (HCK), NCAM2, presenilin-1 (PSEN1), rho-associated protein kinase 2 (ROCK2), RET and sprouty 1 (SPRY1) (Figures 3B and S6G). GO cellular component analysis of enriched *S*-palmitoylated proteins after 7 days of differentiation revealed enrichment in axon, synapse, and neuron projection (Figure 3C). Taken together, these observations point toward an important role of *S*-palmitoylated proteins in neuronal differentiation.^39^

To gain further insights into the dynamics of *S*-palmitoylation during neuronal differentiation and to increase confidence that the proteins detected by the ABE method are bona fide *S*-palmitoylated proteins, LML, an orthogonal method for *S*-palmitoylation profiling was performed (Figure S7A–E).^34^ A 4 h labeling pulse with alk-16 in undifferentiated and differentiated cells at *t* = 0, *t* = 1, and *t* = 7 showed a large overlap in identified *S*-palmitoylated proteins between LML and ABE, with 650 proteins identified by both methods. Of these proteins, about 7% have not been found in previous *S*-palmitoylation studies. Interestingly, the percentage of novel *S*-palmitoylated proteins was higher in differentiated cells, highlighting that cell stimulation can enhance the discovery of new PTM events.

After 1 day of differentiation, a limited number of proteins showed elevated incorporation of alk-16 compared to undifferentiated cells (Figure S7F). However, at *t* = 7, a remarkable elevation of alk-16 incorporation was observed in differentiated cells compared to undifferentiated cells, pointing toward elevated turnover of *S*-palmitoylation in cells stimulated for 7 days (Figure 3D). Interestingly, this trend was not fully reflected in the ABE measurements, which measure steady-state *S*-palmitoylation, as ABE recovers all *S*-acylated proteins while LML likely enriches for *S*-palmitoylated proteins with a high *S*-palmitoylation turnover. These dynamically *S*-palmitoylated proteins may also include low-abundant proteins, which might not be detected with ABE. GO-enrichment analysis (biological process) of the proteins that displayed significantly elevated levels of alk-16 incorporation at *t* = 7 revealed enrichment in processes including exocytosis (of synaptic vesicles), protein *S*-palmitoylation, response to retinoic acid, and axon extension (Figure S7G), highlighting the role of *S*-palmitoylation in this RA-induced neuronal differentiation. Of note, multiple *S*-palmitoylated proteins involved in neurological disorders, for example, β-site APP cleaving enzyme 1 (BACE1), PSEN1, superoxide dismutase 1 (in familial amyotrophic sclerosis), CDC42 (in Schizophrenia), and DNAJC5 (in neuronal ceroid lipofuscinosis) were also identified, indicating that these methods hold great potential to monitor the lipidation of disease-relevant proteins.

A comparison between the HEK293T and SH-SY5Y datasets reveals a high overlap of over 80% between the putative *S*-palmitoylated proteins identified by both ABE and LML in individual cell lines (Figure S6H). The proteins identified by ABE or LML in each cell line exclusively may indicate differences in protein expression or differential regulation of *S*-palmitoylation between the two cell lines. Furthermore, the identification of 378 proteins by both methods in both cell lines provides strong evidence that these proteins are indeed *S*-palmitoylated.

### Comparison of the *S*-Palmitome with the Total Proteome

**Figure 4:**
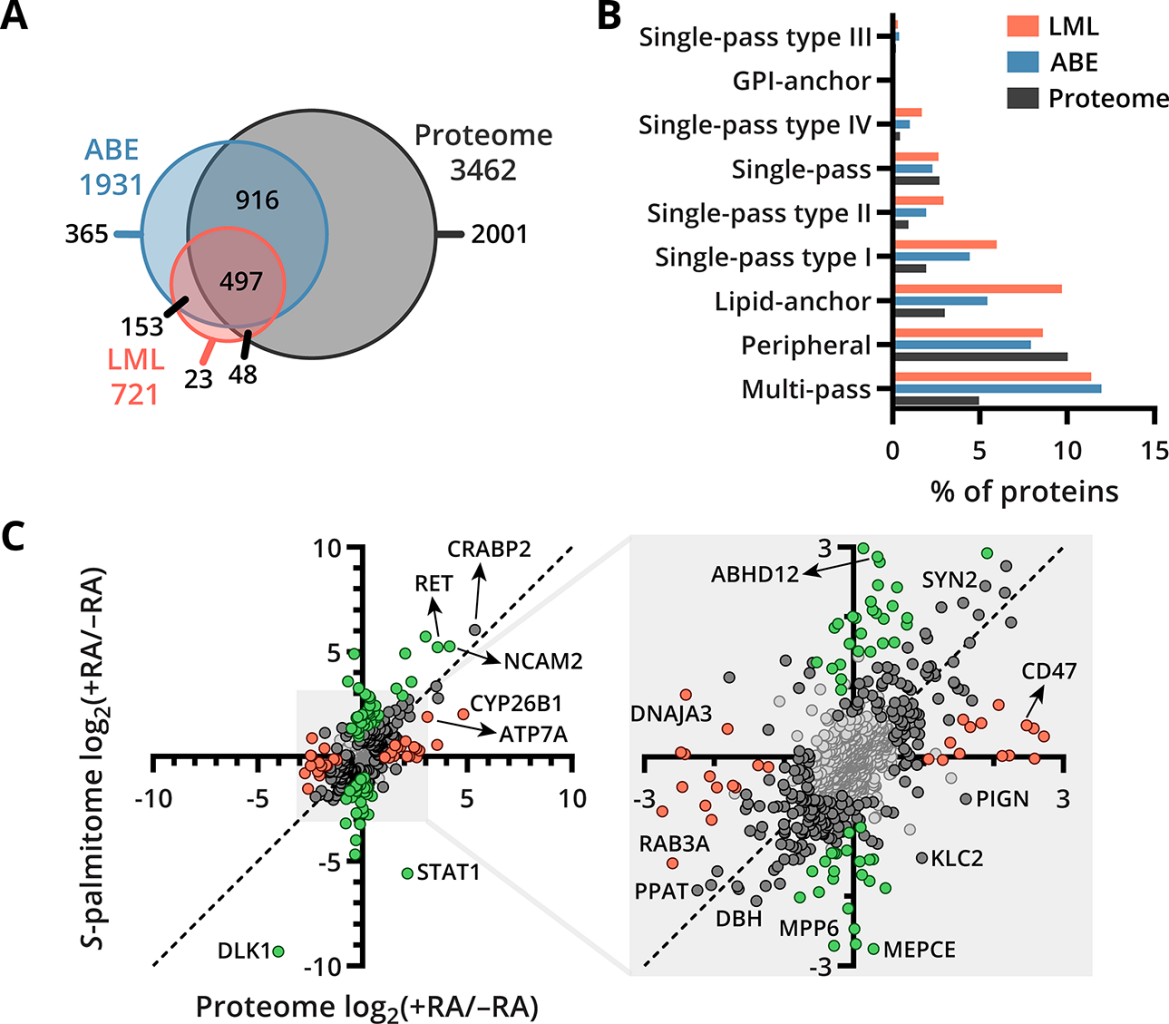
Comparison of the *S*-palmitome and proteome of SH-SY5Y cells during RA-induced neuronal differentiation. (A) Venn diagram showing the overlap in protein identifications by ABE, LML, and total proteome analysis. (B) Bar chart of membrane protein types identified with each method. The membrane protein topology analysis was done with SwissPalm for each method separately. (C) Correlation plot showing the fold changes of the proteins identified by ABE and total proteome analysis. The fold changes are log_2_ LFQ intensities of proteins found in differentiated cells compared to undifferentiated cells at *t* = 7. Proteins with significant changes in their *S*-palmitoylated protein abundance and/or protein abundance are dark gray. To further highlight, proteins with significant changes in *S*-palmitoylated protein abundance are green. Proteins with significant changes in protein abundance are pink. Proteins that did not change significantly are light gray. Dashed line represents equal fold changes (*x* = *y*).

1461 proteins were identified in both the total proteome analysis and *S*-palmitome analysis (Figure 4A). Furthermore, 541 proteins were only identified by *S*-palmitoylation profiling, indicating that the methods for *S*-palmitoylation profiling can detect proteins with relatively low abundance. Analysis of membrane topologies revealed that multi-pass, lipid-anchor, and single-pass type I and II membrane proteins were strongly overrepresented in *S*-palmitoylated proteins (Figure 4B). Remarkably, lipid-anchored proteins constitute almost double the percentage of identified proteins in LML compared to ABE, which points toward high *S*-palmitoylation turnover for these proteins.

To determine whether the changes in *S*-palmitoylated protein abundance are reflected in the protein abundance, the fold changes of differentiated cells to those of the undifferentiated cells from the ABE were compared to the total proteome experiment at *t* = 7 (Figure 4C). The bulk of the proteins cluster along the *x* = *y* diagonal, indicating an equal change in *S*-palmitoylated protein and total protein abundance (e.g., CRABP2). However, several known *S*-palmitoylated proteins, such as CD47, MPP6, and STAT1, deviate from this diagonal, pointing toward a change in *S*-palmitoylation stoichiometry upon RA-induced neuronal differentiation. A spread of smaller differential changes was also observed between *S*-palmitoylated protein abundance and total protein abundance for differentiated cells. These differential changes might also indicate alteration in *S*-palmitoylation stoichiometry; however, the development of more precise *S*-palmitoylation stoichiometry measurement strategies is required to uncover if these differences originate from changing *S*-palmitoylation stoichiometry or analytical variability. In addition, we normalized the ABE data to the total proteome data to gain insight into proteins that show a relatively high intensity in the ABE analysis compared to their protein abundance. Multiple guanine nucleotide-binding proteins show a relatively high intensity in the ABE analysis compared to the total proteome in both RA-treated and untreated SH-SY5Y cells, which points toward a high *S*-acylation stoichiometry for this protein class (Table S2).

**Figure 5:**
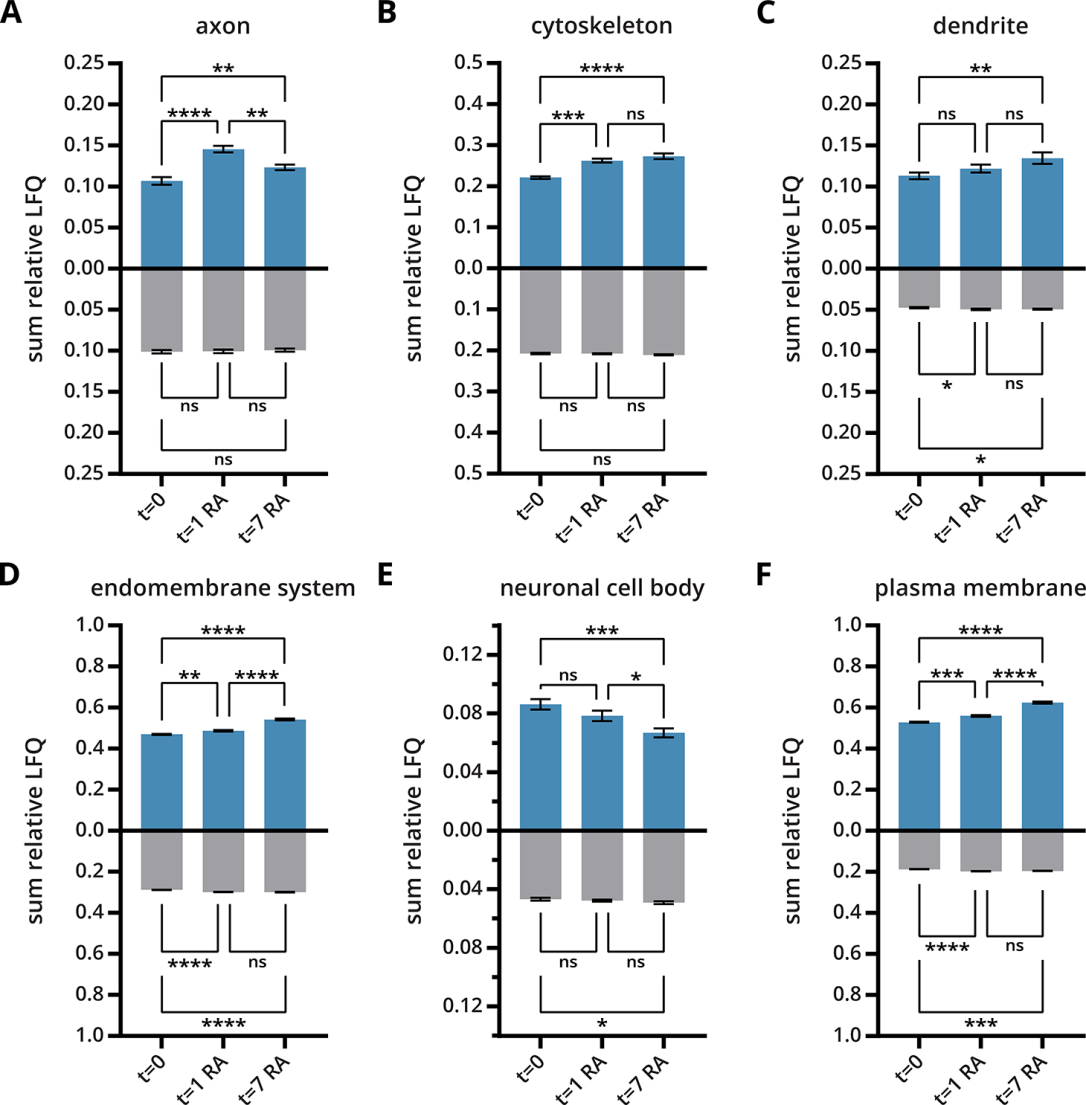
Comparison of the *S*-palmitome and proteome of SH-SY5Y cells during RA-induced neuronal differentiation. (A–F) Bar charts show the sum of relative LFQ intensities of *S*-acylated proteins linked to a specific subcellular localization identified during RA-induced neuronal differentiation (*n* = 3, blue = ABE method, gray = total proteome). The adjusted *p*-value was calculated using an ordinary one-way ANOVA test employing Tukey’s multiple comparisons test to determine significant differences between the three conditions (ns = not significant; *p ≤ 0.0332; **p ≤ 0.0021; ***p ≤ 0.0002; and ****p ≤ 0.0001). Data were mean ± SD.

*S*-palmitoylation is involved in protein trafficking and can target proteins to specific organelles or cellular compartments.^40^ To gain insight into the localization of *S*-palmitoylated proteins, we analyzed the differences in bulk *S*-palmitoylated protein abundances linked to specific cellular locations between differentiated and undifferentiated cells.^20^ Increases in summed *S*-palmitoylated protein abundance in differentiated cells compared to undifferentiated cells were observed for proteins localized in the axon/dendrite, cytoskeleton, endomembrane system, and plasma membrane, while a decrease was observed for the neuronal cell body (Figure 5A–F). Importantly, the summed total protein abundance in these organelles remained constant (Figure 5A–F). These observations indicate the importance of *S*-palmitoylation in protein localization during neurite outgrowth, which involves two coordinated mechanisms: (1) the rearrangement of the cytoskeleton and (2) the expansion of the plasma membrane by exo- and endocytosis. Additionally, an increase in bulk *S*-palmitoylated protein abundance was observed for organellar membranes, including the lysosomal membrane, endoplasmic reticulum (ER) membrane, Golgi membrane, and nuclear membrane (Figure S8A–D).

### *S*-Palmitoylation of the Exocytosis Regulatory Machinery during Neuronal Differentiation

**Figure 6:**
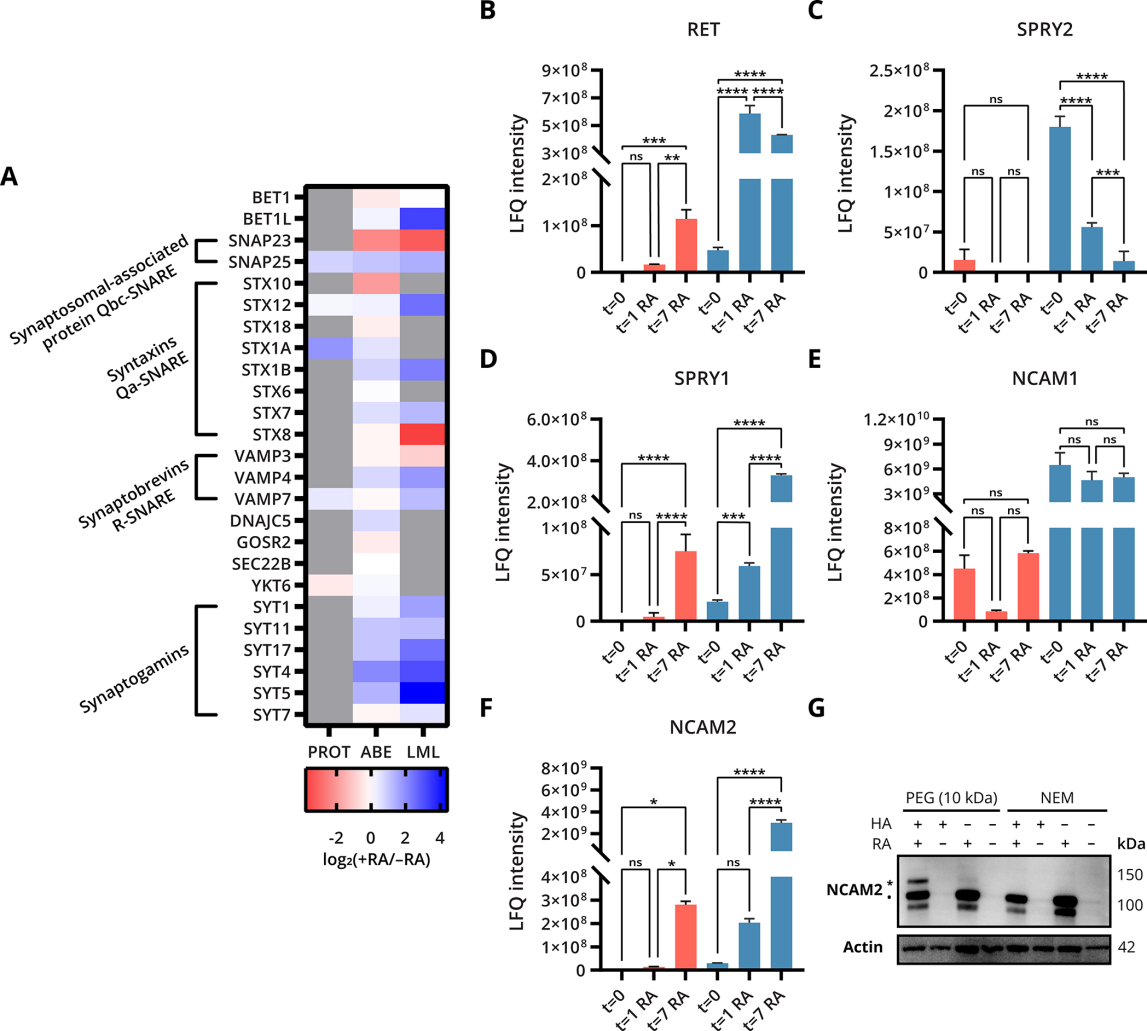
*S*-palmitoylated proteins in RA-induced neuronal differentiation of SH-SY5Y cells. (A) Heatmap of the fold changes of proteins involved in exocytotic vesicle fusion in differentiated cells compared to undifferentiated cells at *t* = 7 (PROT = proteome). Proteins not detected by a method are gray. (B–F) Bar chart illustrates the LFQ intensity of (B) RET, proto-oncogene tyrosine-protein kinase receptor; (C) SPRY2, sprouty 2; (D) SPRY1, sprouty 1; (E) NCAM1, neural cell adhesion molecule 1; and (F) NCAM2, neural cell adhesion molecule 2 during RA-induced neuronal differentiation (*n* = 3, pink = LML method, blue = ABE method). The adjusted *p*-value was calculated using an ordinary one-way ANOVA test employing Tukey’s multiple comparisons test to determine significant differences between the three conditions (ns = not significant; *p ≤ 0.0332; **p ≤ 0.0021; ***p ≤ 0.0002; and ****p ≤ 0.0001). Data were mean ± SD. (G) Western blot shows the acyl-PEG exchange assay, detecting palmitoylation-dependent mobility shift of NCAM2 in differentiated cells at *t* = 7. NCAM2 was labeled with mPEG-10k, and samples were analyzed using anti-NCAM2 antibody. ● Refers to non-PEGylated NCAM2. * Refers to a single PEGylation event.

Neuronal differentiation is accompanied by expansion of the plasma membrane surface area as axons are extended and neurons polarize and grow.^41^ One of the key processes for the delivery of essential components to the plasma membrane is exocytotic vesicle fusion, which is driven by soluble NSF (*N*-ethylmaleimide-sensitive factor) attachment protein receptor (SNARE) proteins.^42^ SNARE proteins have been implicated in several critical neuronal functions involving exocytotic vesicle fusion, including synaptic transmission, neurite outgrowth, axon specification, axon extension, and synaptogenesis.^43^ Importantly, SNARE proteins such as SNAP25, VAMP2, and syntaxin-1 are known to be *S*-palmitoylated, and the post-translational modification likely plays a key role in SNARE-mediated vesicle fusion.^44^ We identified *S*-palmitoylation of multiple key components of the SNARE-mediated synaptic vesicle fusion machinery during neuronal differentiation (Figure 6A). *S*-palmitoylation of multiple syntaxins was detected by both LML and ABE. Interestingly, incorporation of alk-16 in multiple syntaxins (STX1B, STX7, and STX12) was elevated in differentiated cells compared to undifferentiated cells after 7 days of RA stimulation. This effect was not reflected in the ABE measurements, pointing toward a higher alk-16 turnover on these syntaxins in differentiated cells. Levels of *S*-palmitoylated SNAP23 decreased as measured by both ABE and LML at *t* = 7, while SNAP25 had increased slightly in both ABE and LML. The *S*-palmitoylation of cysteine string protein (DNAJC5), a co-chaperone of SNAP25, also increased at *t* = 7 in the ABE measurement. In addition, intracellular Ca^2+^ is a stimulus for vesicle fusion, which is sensed by membrane trafficking proteins synaptogamins, and affects vesicle fusion by Ca^2+^-dependent or -independent interactions with SNARE proteins.^45,46^ Elevated levels of a subset of *S*-palmitoylated synaptogamins (SYT4, SYT5, and SYT17) were detected by both ABE and LML, while levels of *S*-palmitoylated synaptogamins SYT1 and SYT7 remained stable. Notably, many of these proteins were only identified by *S*-palmitoylation profiling and not in the total proteome analysis. Together, these data show that many components of the SNARE-mediated vesicle fusion machinery are *S*-palmitoylated during neuronal differentiation, and a subset undergoes changes in the levels of *S*-palmitoylated protein upon neuronal differentiation.

### *S*-Palmitoylation of the Receptor Tyrosine Kinase RET

One of the proteins which showed the highest elevation in both levels of *S*-palmitoylated protein and incorporation of alk-16 was the proto-oncogene tyrosine-protein kinase receptor ret (RET). This protein was not known to be *S*-palmitoylated, but here we show *S*-palmitoylation of RET by two orthogonal methods (Figure 6B). RET is involved in the regulation of sensory neuron dendrite growth and integrin-mediated adhesion, in which its *S*-palmitoylation state may play a role by controling RET activation and downstream signaling.^47−49^ Mutations that either activate or inhibit RET function can result in several disorders, such as cancer (e.g., multiple endocrine neoplasia type 2) and Hirschsprung disease. Moreover, several members of the sprouty homolog family that are regulators of RET downstream signaling were found to be *S*-palmitoylated in SH-SY5Y cells as well. Sprouty-2 (SPRY2) was found to be palmitoylated on 10 sites (Supporting Information 2) and is a negative feedback regulator of several receptor tyrosine kinases (RTKs), including RET.^50^ The decreased levels of *S*-palmitoylated SPRY2, observed by both ABE and LML, may contribute to elevated RET signaling during neuronal differentiation (Figure 6C). In addition, SPRY1 was found to be *S*-palmitoylated on Cys181 and 184 after 7 days of differentiation (Supporting Information 2). In contrast to SPRY2, SPRY1, which is also known as an antagonist for RET, significantly increased in *S*-palmitoylated protein abundance as determined by both ABE and LML assays (Figure 6D).^51^ SPRY1 specifically inhibits the RAS-ERK/MAPK downstream signaling of RET, thus increasing and decreasing *S*-palmitoylation on SPRY1 and SPRY2, respectively, may tune RET downstream signaling.^51,52^ SPRY4 was detected to be *S*-palmitoylated on Cys138, 159 and 162 (Supporting Information 2). Levels of *S*-palmitoylated SPRY4 or sprouty-related EVH1 domain-containing protein 2 (SPRED2), which are negative regulators of the Ras/Raf/ERK/MAPK pathway, showed small differences or did not change upon neuronal differentiation (Figure S9A,B).

### *S*-Palmitoylation of Neural Cell Adhesion Molecules

Neural cell adhesion molecule 1 (NCAM1) and neural cell adhesion molecule 2 (NCAM2) belong to the cell adhesion molecules of the immunoglobulin superfamily. NCAM1 has been shown to play a fundamental role in neurite development, neuronal migration, and synaptogenesis.^53^ NCAM2 was recently also found to regulate neurite outgrowth and synapse formation.^54^ Interestingly, at *t* = 0, levels of *S*-palmitoylated NCAM1 exceed the levels of *S*-palmitoylated NCAM2 as determined by ABE (Figure 6E,F). However, levels of *S*-palmitoylated NCAM2 are already significantly elevated after 1 day of differentiation and are increased ∼100 fold after 7 days of differentiation, suggesting a role of *S*-palmitoylated NCAM2 in neuronal differentiation. Increased NCAM2 protein levels upon RA-induced neuronal differentiation were confirmed by western blot (Figure S9C), and acyl-PEG exchange showed that the *S*-palmitoylation stoichiometry of NCAM2 after 7 days of differentiation was ∼10% (Figure 6G). NCAM2 was found to be *S*-palmitoylated at Cys42, 93, 281, and 380 (Supporting Information 2). In addition to NCAM1 and NCAM2, lipid metabolic labeling also indicated increased alk-16 incorporation on neural cell adhesion molecule L1 (L1CAM) after 7 days of differentiation (Figure S9E). Interestingly, levels of *S*-palmitoylated neuronal cell adhesion molecule (NrCAM) showed an opposite trend and were already reduced after 1 day of differentiation (Figure S9D). Taken together, these data indicate that multiple members of the cell adhesion molecules of the immunoglobulin superfamily that are known to play a role in neuronal differentiation change in *S*-palmitoylated protein abundance. Understanding the role of *S*-palmitoylation on neural cell adhesion molecules warrants further investigation of these PTM events.

## Conclusions

ABE and LML were applied to study *S*-palmitoylation during RA-induced neuronal differentiation of SH-SY5Y cells. Performing ABE and LML in parallel allowed assignment of a subset (650) of high confidence *S*-palmitoylated proteins. In addition, chloroacetamide labeling of released cysteines in the acyl-biotin exchange approach allows simultaneous profiling of *S*-palmitoylated proteins and the identification of *S*-palmitoylation sites. By identification of 2002 *S*-palmitoylated proteins and 475 *S*-palmitoylation sites, this study provides a rich resource for gaining insight into the role of *S*-palmitoylation in the CNS. Moreover, *S*-palmitoylation analysis of RA-stimulated SH-SY5Y cells revealed (1) changes in abundance of *S*-palmitoylated proteins during neuronal differentiation; (2) increased *S*-palmitoylation turnover as determined by LML after 7 days of RA stimulation; and (3) discovery of new *S*-palmitoylated proteins. The *S*-palmitoylation analysis indicates a role for *S*-palmitoylation in several processes/protein classes that are known to be important for neuronal differentiation, including RET signal transduction, SNARE-mediated exocytosis, and neural cell adhesion molecules.

Overall, *S*-palmitoylation profiling by employing ABE and LML in parallel during RA-induced differentiation of SH-SY5Y cells revealed a substantial overlap in identified proteins by both methods and suggests an important role for *S*-palmitoylation in neuronal differentiation.

## Supporting Information Available

Advantages and disadvantages of LML and ABE; *S*-acylated proteins and sites identified by ABE in HEK293T cells; *S*-palmitoylated proteins identified by LML in HEK293T cells; morphology and proteome changes in SH-SY5Y cells during RA-induced neuronal differentiation; protein abundances of *S*-acyl protein thioesterases in RA-induced neuronal differentiation of SH-SY5Y cells; *S*-acylated proteins identified by ABE in SH-SY5Y cells; *S*-palmitoylated proteins identified by LML in SH-SY5Y cells; comparison of the *S*-palmitome and proteome of SH-SY5Y cells during RA-induced neuronal differentiation; *S*-palmitoylated proteins in RA-induced neuronal differentiation of SH-SY5Y cells; and western blots of acyl-PEG exchange assay with NCAM2 in SH-SY5Y cells at *t* = 7 (PDF)
*S*-Palmitoylation protein and site identification in HEK293T (XLSX)
*S*-Palmitoylation protein and site identification in SH-SY5Y (XLSX)

## Notes

The mass spectrometry proteomics data have been deposited to the ProteomeXchange Consortium via the PRIDE partner repository with the dataset identifier PXD040613.

## Abbreviations

ABE: acyl-biotin exchange
alk-16: 15-hexadecynoic acid
ABHD17A-C: α/β hydrolase domain containing protein 17A-C
AD: Alzheimer’s disease
APP: amyloid-beta precursor protein
APT1/2: acyl-protein thioesterase 1 and 2
BACE1: beta-secretase1
BCA: bicinchoninic acid
CAA: chloroacetamide
CNS: central nervous system
CRABP2: cellular retinoic acid binding protein 2
CuAAC: copper-catalyzed alkyne–azide click reaction
CYP26B1: cytochrome P450 family 26 subfamily B member 1
DMEM: Dulbecco’s modified Eagle medium
DNAJC5: DnaJ heat shock protein 40 homolog
DPBS: Dulbecco’s phosphate-buffered saline
DTDP: 4,4′-dithiodipyridine
ER: endoplasmic reticulum
FALS: familial amyotrophic sclerosis
FBS: fetal bovine serum
GO: gene ontology
HA: hydroxylamine
HD: Huntington’s disease
HTT: Huntingtin
LC–MS/MS: liquid chromatography–tandem mass spectrometry
LFQ: label-free quantification
LML: lipid metabolic labeling
NCAM: neural cell adhesion molecule
NCSTN: nicastrin
NEM: *N*-ethyl maleimide
PATs: *S*-palmitoyl transferases
PPT1: palmitoyl protein thioesterase 1
RA: retinoic acid
RET: proto-oncogene tyrosine-protein kinase receptor
SNARE: soluble *N*-ethyl maleimide sensitive factor attachment protein receptor
SOD1: superoxide dismutase 1
SPRY: sprouty
TCEP: tris(2-carboxyethyl)phosphine hydrochloride
ZDHHCs: *S*-palmitoyl transferases
