# Supplementary material for: S-Palmitoylation during Retinoic Acid-Induced Neuronal Differentiation of SH-SY5Y Neuroblastoma Cells
Source: J Proteome Res. 2023 Jun 9;22(7):2421–35. doi: 10.1021/acs.jproteome.3c00151 (PMC10337253; doi:10.1021/acs.jproteome.3c00151)
Supplement: Supplementary file 1 — pr3c00151_si_001.pdf [file pr3c00151_si_001.pdf]

Supporting information

S-palmitoylation during retinoic acid induced neuronal differentiation of SH-SY5Y neuroblastoma cells

Samiksha Sardana[1,2], *Anneroos E. Nederstigt*[1,2], *Marc P. Baggelaar*[1,2,*]

[1.] Biomolecular Mass Spectrometry and Proteomics, Bijvoet Center for Biomolecular Research and Utrecht Institute for Pharmaceutical Sciences, University of Utrecht, Padualaan 8, Utrecht 3584 CH, The Netherlands

[2.] Netherlands Proteomics Center, Padualaan 8, Utrecht 3584 CH, The Netherlands

[*] Contact details for correspondence: m.p.baggelaar@uu.nl

Supporting figures

Figure S1. The advantages and disadvantages of LML and ABE.

Figure S2. *S*-acylated proteins and sites identified by ABE in HEK293T cells.

Figure S3. *S*-palmitoylated proteins identified by LML in HEK293T cells.

Figure S4. Morphology and proteome changes in SH-SY5Y cells during RA-induced neuronal differentiation.

Figure S5. Protein abundances of *S*-acyl protein thioesterases in RA-induced neuronal differentiation of SH-SY5Y cells.

Figure S6. *S*-acylated proteins identified by ABE in SH-SY5Y cells.

Figure S7. *S*-palmitoylated proteins identified by LML in SH-SY5Y cells.

Figure S8. Comparison of the *S*-palmitome and proteome of SH-SY5Y cells during RA-induced neuronal differentiation.

Figure S9. *S*-palmitoylated proteins in RA-induced neuronal differentiation of SH-SY5Y cells.

Figure S10. Western blots of acyl-PEG exchange assay with NCAM2 in SH-SY5Y cells at t=7.

Supplementary data S1. *S*-palmitoylation protein and site identification in HEK293T (XLSX)

Supplementary data S2. *S*-palmitoylation protein and site identification in SH-SY5Y (XLSX)

Method	Advantages	Disadvantages
Lipid metabolic labelling	▪ Allows profiling of (new and dynamic) *S*-palmitoylated proteins<br>▪ Allows fatty acid specificity<br>▪ Allows in-gel visualisation<br>▪ Compatible with live-cell microscopy<br>▪ Compatible with pulse-chase studies	▪ Not applicable in tissues; only *in vitro* studies<br>▪ Endogenously biotinylated proteins are false positives<br>▪ Fatty acid probe can be metabolised (false positives)<br>▪ Does not provide information on the *S*-palmitoylation site
Acyl-biotin exchange	▪ Allows large-scale profiling and quantitative analysis of *S*-acylated proteins<br>▪ Compatible with tissues	▪ Substantial sample loss due to the number of reaction and purification steps<br>▪ Incomplete blockade of free cysteines can result in false positives<br>▪ Incomplete thioester hydrolysis and/or biotin labelling can result in false negatives<br>▪ Enzymes with a thioester intermediate are false positives<br>▪ Endogenously biotinylated proteins are false positives<br>▪ Does not provide information on the *S*-palmitoylation site

Supporting Figure 1. The advantages and disadvantages of lipid metabolic labelling and acyl-biotin exchange are provided in the table.

A

B

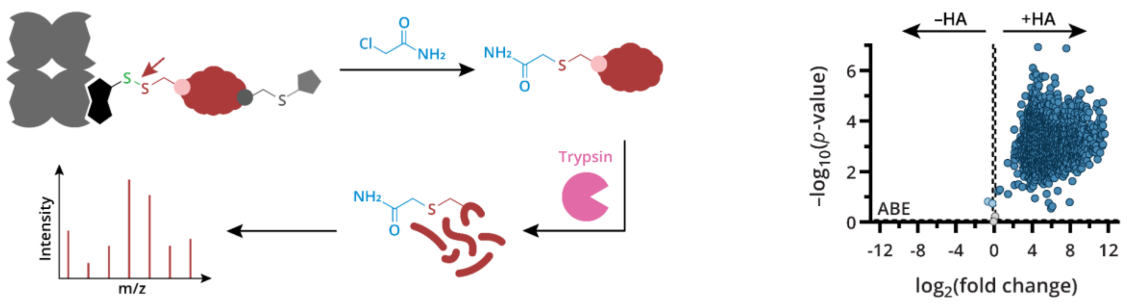

C

PAT	SiteID by ABE	Active site
ZDHHC3	143	157
ZDHHC5	**236**, **237**, 245	134
ZDHHC6	**328**, **329**, **343**	129
ZDHHC8	236, 237, 245	134
ZDHHC12	102	127
ZDHHC16	174, 177	185
ZDHHC18	339, 340, 344	222
ZDHHC20	263	156

D

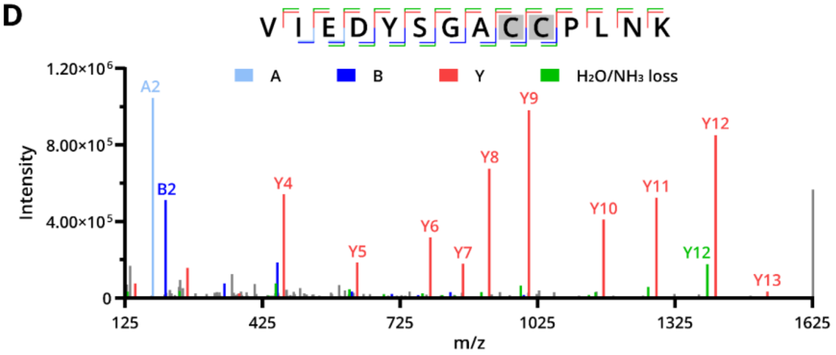

Supporting Figure 2. (A) In the ABE method, after elution of *S*-acylated proteins with TCEP, the free cysteines are labelled with chloroacetamide to identify the *S*-acylation site-containing peptides with mass spectrometry. (B) Volcano plot of the LFQ proteomics analysis of *S*-acylated proteins in HEK293T cells enriched with ABE workflow (+/-HA). Putative *S*-acylated proteins are dark blue. Dashed line represents Student's unpaired *t*-test significance cut-off (FDR 0.01, S0 0.5 and *n*=2 biological replicates). (C) Table shows the *S*-palmitoylation and active sites of protein *S*-palmitoyl transferases. The sites in bold have been validated in previous *S*-palmitoylation studies. (D) MS/MS spectrum shows the site-containing peptide of ZDHHC6 from the ABE method (Y-ions = red, A-ions = light blue, B-ions = dark blue, and H2O/NH3 losses = green).

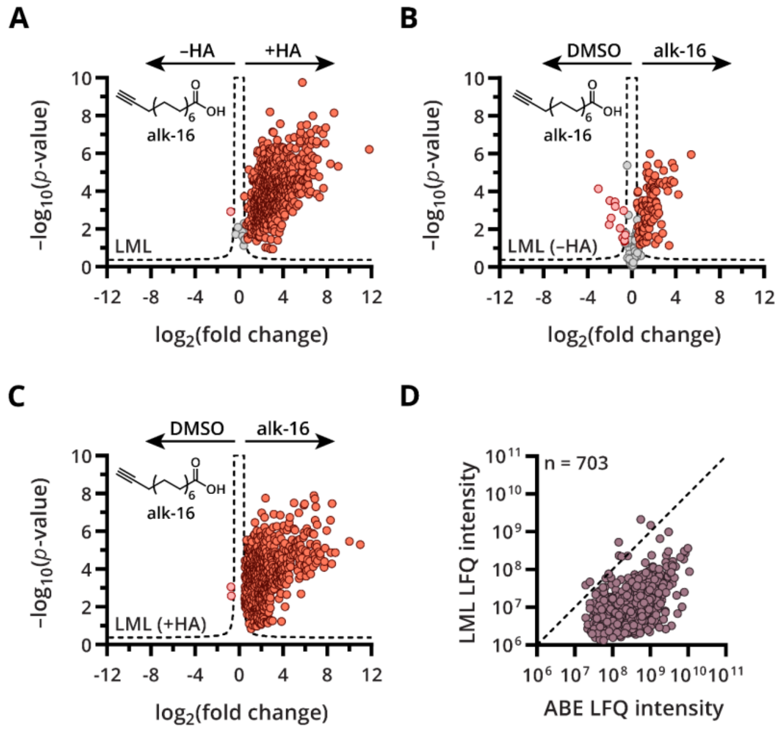

Supporting Figure 3. (A) Volcano plot of the LFQ proteomics analysis of alk-16-labelled proteins in HEK293T cells enriched with LML workflow (+/- HA). Putative alk-16-labelled proteins identified are orange. Dashed line represents Student's unpaired *t*-test significance cut-off (FDR 0.01, S0 0.5 and n=3 technical replicates). (B) Volcano plot of the LFQ proteomics analysis of proteins in HEK293T cells enriched with LML workflow (without HA). Dashed line represents Student's unpaired *t*-test significance cut-off (FDR 0.01, S0 0.5 and n=3 technical replicates). (C) Volcano plot of the LFQ proteomics analysis of proteins in HEK293T cells enriched with LML workflow (with HA). Data points in orange are potential alk-16-labelled proteins in alk-16-treated compared to DMSO-treated samples. Dashed line represents Student's unpaired *t*-test significance cut-off (FDR 0.01, S0 0.5 and n=3 technical replicates). (D) Correlation plot showing the LFQ intensities of the common proteins identified by ABE and LML method. Black line indicates equal intensities (x = y).

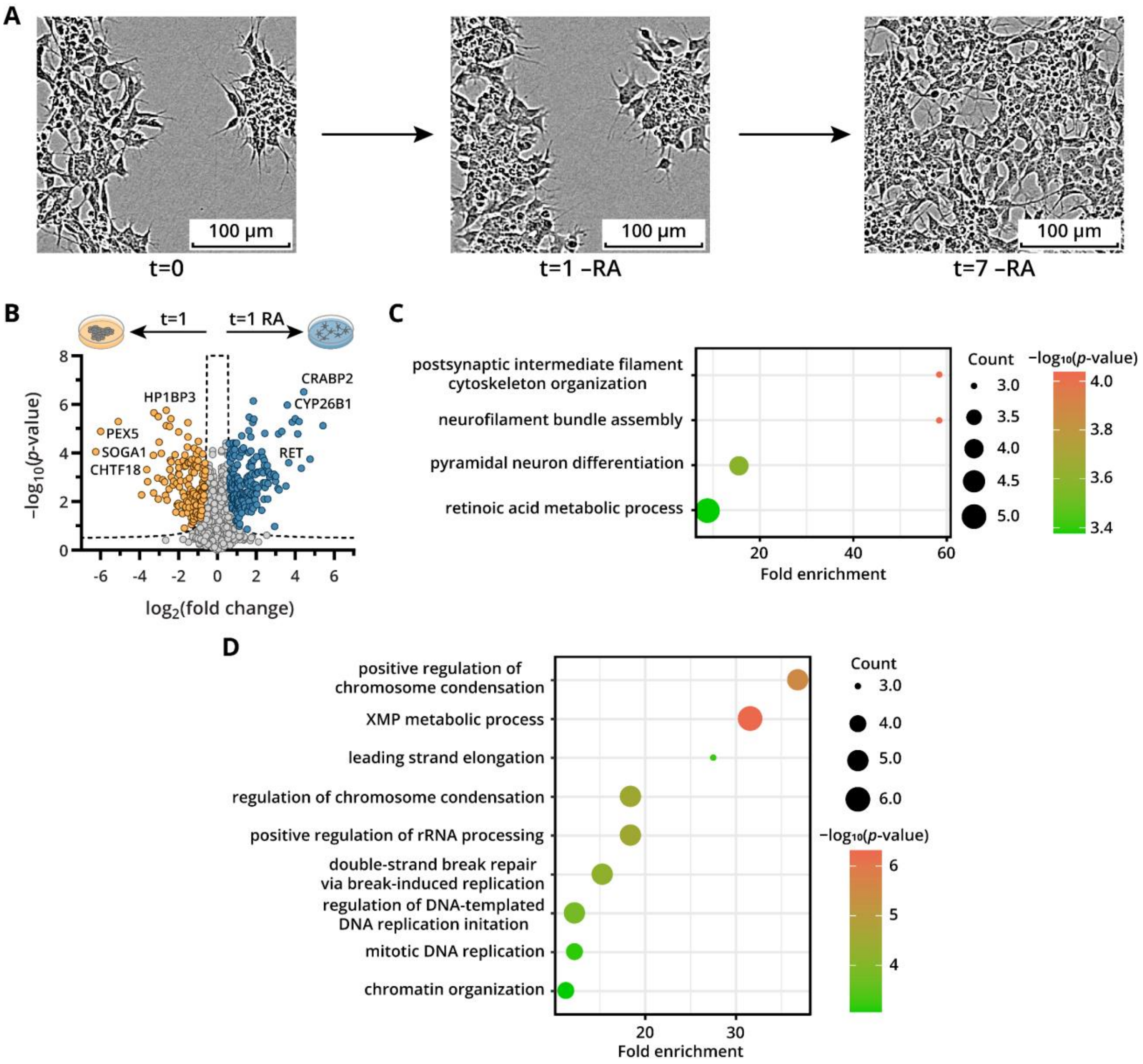

Supporting Figure 4. (A) Phase contrast microscopy images of SH-SY5Y cells without RA treatment over a 7-day period (magnification: 20x). (B) Volcano plot of the LFQ proteomics analysis of proteins in SH-SY5Y cells at t=1 (+/- RA). Proteins downregulated during differentiation are orange and upregulated proteins are blue. Dashed line represents Student's unpaired *t*-test significance cut-off (FDR 0.01, S0 0.5, and *n*=3 biological replicates). (C) GO biological process analysis for upregulated proteins at t=7 (FDR < 0.05). (D) GO biological process analysis for downregulated proteins at t=7 (FDR < 0.05).

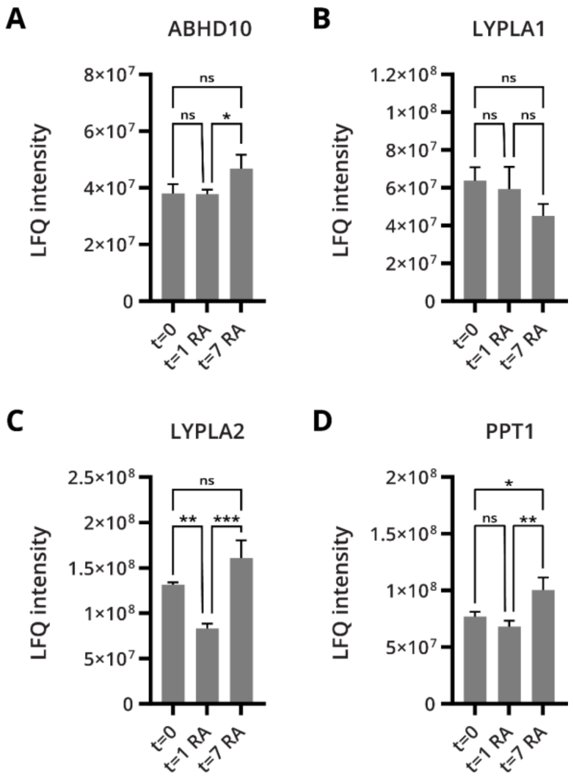

Supporting Figure 5. (A-D) Bar chart illustrates the LFQ intensity of (A) ABHD10, α/β hydrolase domain-containing protein 10; (B) LYPLA1, acyl-protein thioesterase 1; (C) LYPLA2, acyl-protein thioesterase 2; and (D) PPT1, palmitoyl-protein thioesterase 1 during RA-induced neuronal differentiation (*n*=3, total proteome analysis). The adjusted *p*-value was calculated using ordinary one-way ANOVA test employing Tukey's multiple comparisons test to determine significant differences between the three conditions (ns = not significant; *$p \leq 0.0332$; **$p \leq 0.0021$; ***$p \leq 0.0002$; ****$p \leq 0.0001$). Data were mean ± SD.

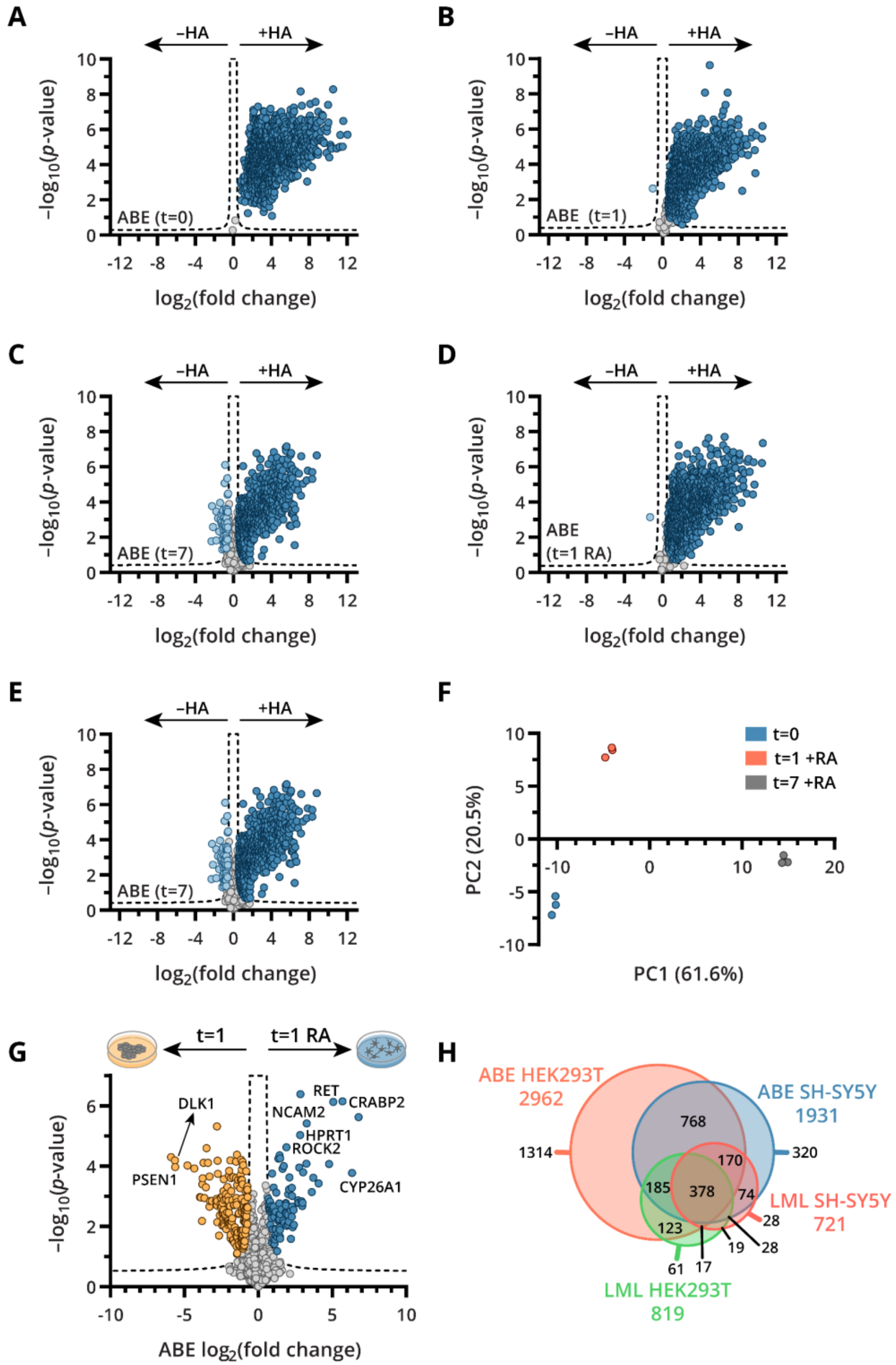

Supporting Figure 6. (A-E) Volcano plot of the LFQ proteomics analysis of *S*-acylated proteins in SH-SY5Y cells enriched with ABE workflow in five conditions (+/- HA). Putative *S*-acylated proteins are dark blue. Dashed line represents Student's unpaired *t*-test significance cut-off (FDR 0.01, S0 0.5 and *n*=3 biological replicates). (F) PCA

score plot of the LFQ proteomics analysis of the 1151 *S*-acylated proteins found in all three time points of RA-induced neuronal differentiation. (G) Volcano plot of the LFQ proteomics analysis of *S*-acylated proteins in SH-SY5Y cells at t=1 enriched with ABE workflow (+/- RA). Proteins decreased in *S*-palmitoylated protein abundance during differentiation are orange and proteins increased are blue. Dashed line represents Student's unpaired *t*-test significance cut-off (FDR 0.01, S0 0.5, and *n*=3 biological replicates). (H) Venn diagram showing the overlap in *S*-palmitoylated protein identifications by ABE and LML in HEK293T and SH-SY5Y cells.

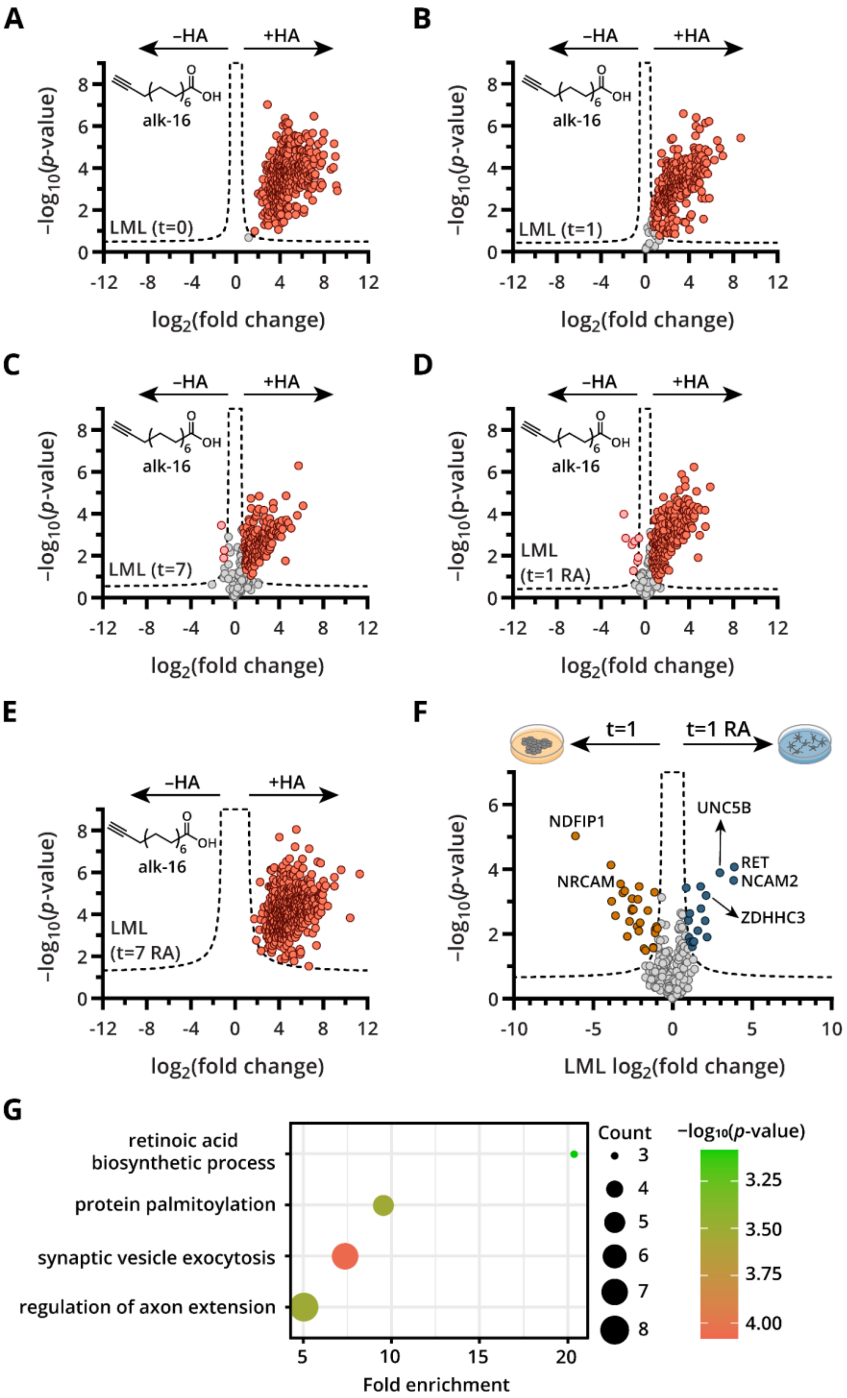

Supporting Figure 7. (A-E) Volcano plot of the LFQ proteomics analysis of *S*-acylated proteins in SH-SY5Y cells enriched with LML workflow in five conditions (+/- HA). Putative *S*-acylated proteins are orange. Dashed line represents Student's unpaired *t*-test significance cut-off (FDR 0.01, S0 0.5 and *n*=3 biological replicates). (F) Volcano plot of the LFQ proteomics analysis of *S*-acylated proteins in SH-SY5Y cells at t=1 enriched with LML workflow (+/- RA). Proteins decreased in *S*-palmitoylated protein abundance during differentiation are orange and proteins increased

are blue. Dashed line represents Student's unpaired *t*-test significance cut-off (FDR 0.01, S0 0.5, and *n*=3 biological replicates). (G) GO biological process analysis for proteins increased in *S*-palmitoylated protein abundance at t=7 (FDR $<$ 0.05, LML).

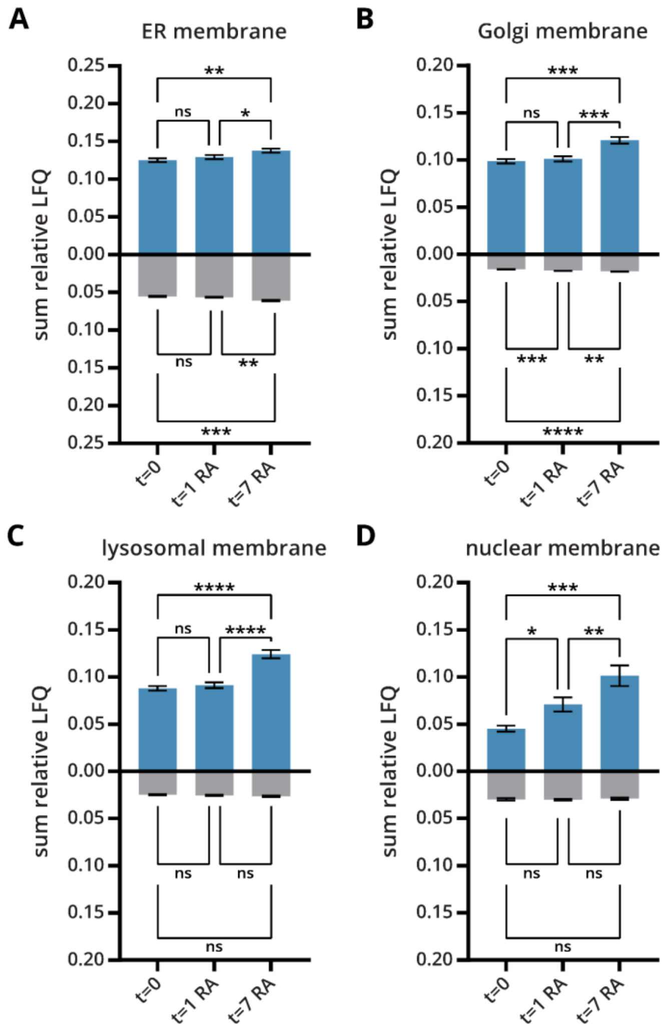

Supporting Figure 8. (A-D) Bar chart shows the sum of relative LFQ intensities of *S*-acylated proteins linked to a specific subcellular localisation identified during RA-induced neuronal differentiation (*n*=3, blue = ABE method, grey = total proteome). The adjusted *p*-value was calculated using ordinary one-way ANOVA test employing Tukey's multiple comparisons test to determine significant differences between the three conditions (ns = not significant; *$p \leq 0.0332$; **$p \leq 0.0021$; ***$p \leq 0.0002$; ****$p \leq 0.0001$). Data were mean ± SD.

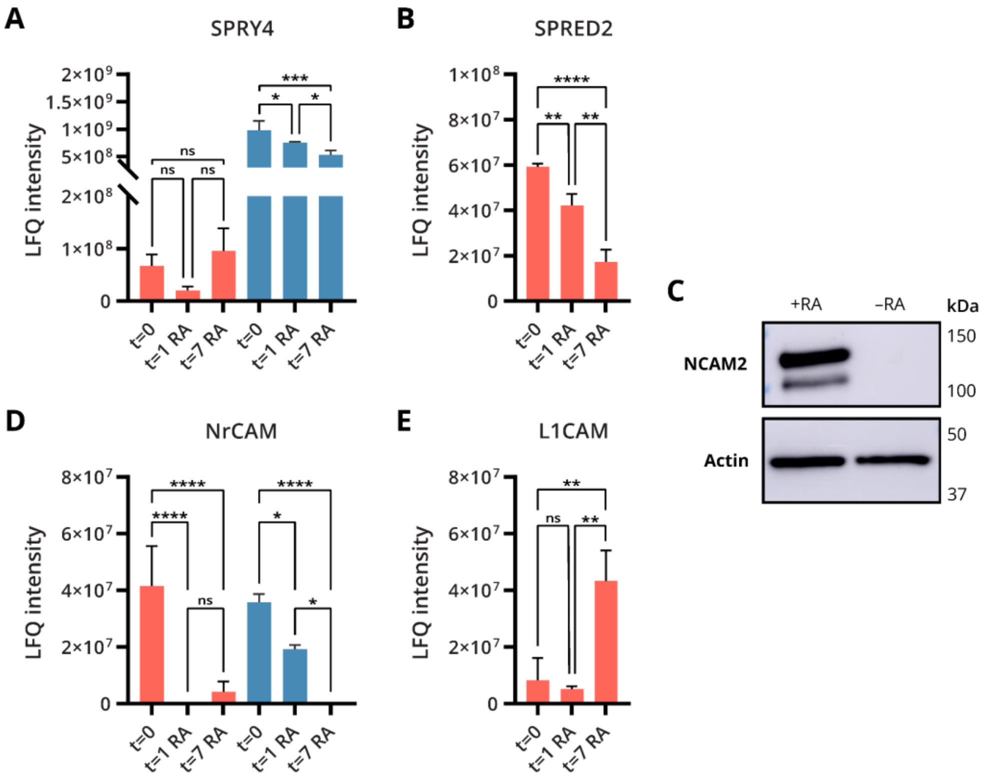

Supporting Figure 9. (A-B) Bar chart illustrates the LFQ intensity of (A) SPRY4, sprouty 4; and (B) SPRED2, sprouty-related EVH1 domain-containing protein 2 during RA-induced neuronal differentiation (*n*=3, pink = LML method, blue = ABE method). The adjusted *p*-value was calculated using ordinary one-way ANOVA test employing Tukey's multiple comparisons test to determine significant differences between the three conditions (ns = not significant; *$p \leq 0.0332$; **$p \leq 0.0021$; ***$p \leq 0.0002$; ****$p \leq 0.0001$). Data were mean ± SD. (C) Western blot shows NCAM2 expression levels in differentiated and undifferentiated cells at t=7. The samples were analysed using anti-NCAM2 antibody. (D-E) Bar chart illustrates the LFQ intensity of (D) NrCAM, neuronal cell adhesion molecule; and (E) L1CAM, neural cell adhesion molecule L1 during RA-induced neuronal differentiation (*n*=3, pink = LML method, blue = ABE method). The adjusted *p*-value was calculated using ordinary one-way ANOVA test employing Tukey's multiple comparisons test to determine significant differences between the three conditions (ns = not significant; *$p \leq 0.0332$; **$p \leq 0.0021$; ***$p \leq 0.0002$; ****$p \leq 0.0001$). Data were mean ± SD.

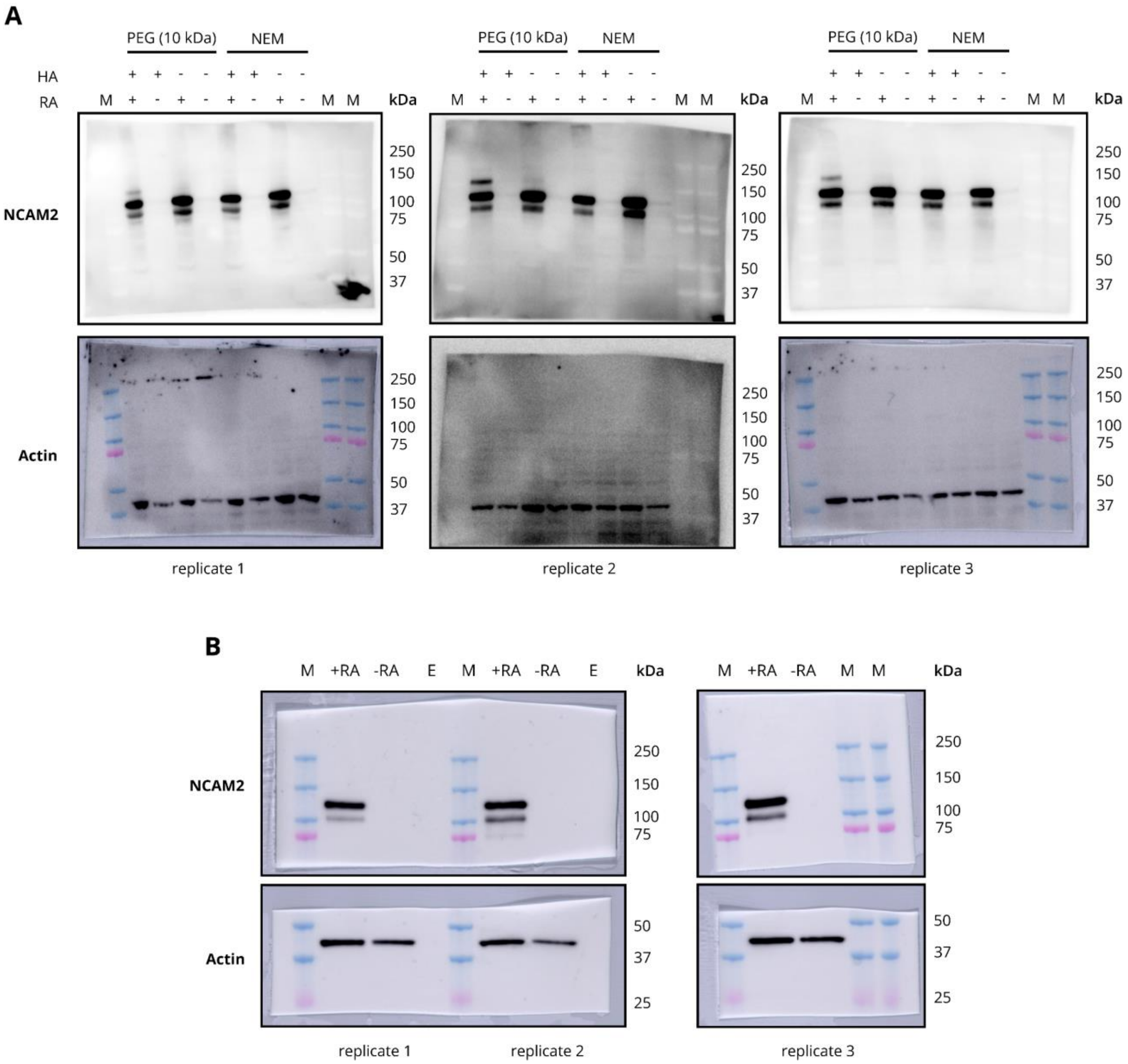

Supplementary Figure 10. (A) Western blots show the acyl-PEG exchange assay, detecting palmitoylation-dependent mobility shift of NCAM2 in differentiated cells at t=7. NCAM2 was labelled with mPEG-10k and samples were analysed using anti-NCAM2 antibody. (B) Western blots show NCAM2 expression levels in differentiated and undifferentiated cells at t=7. The samples were analysed using anti-NCAM2 antibody.
